# How individuals change language

**DOI:** 10.1371/journal.pone.0252582

**Published:** 2021-06-02

**Authors:** Richard A. Blythe, William Croft

**Affiliations:** 1 SUPA, School of Physics and Astronomy, University of Edinburgh, Edinburgh, United Kingdom; 2 Department of Linguistics, University of New Mexico, Albuquerque, New Mexico, United States of America; Leiden University, NETHERLANDS

## Abstract

Languages emerge and change over time at the population level though interactions between individual speakers. It is, however, hard to directly observe how a single speaker’s linguistic innovation precipitates a population-wide change in the language, and many theoretical proposals exist. We introduce a very general mathematical model that encompasses a wide variety of individual-level linguistic behaviours and provides statistical predictions for the population-level changes that result from them. This model allows us to compare the likelihood of empirically-attested changes in definite and indefinite articles in multiple languages under different assumptions on the way in which individuals learn and use language. We find that accounts of language change that appeal primarily to errors in childhood language acquisition are very weakly supported by the historical data, whereas those that allow speakers to change incrementally across the lifespan are more plausible, particularly when combined with social network effects.

## Introduction

Human language is a multiscale phenomenon. A language is shared by a large population, that is, the speech community: it is a set of linguistic conventions, characteristic of the population as a whole. Yet language originates in individuals. Individuals in a population use language to achieve specific communicative goals, and through repeated interactions there emerge the linguistic conventions of the speech community. These conventions also change over time, and as speech communities split, the linguistic conventions of the speech communities diverge, leading to variation across languages.

How does the behaviour of individual speakers lead to change in linguistic conventions and ultimately the emergence of linguistic diversity? It transpires that this is one of the most debated questions in the study of language change for at least a century [1]. A widely-held view is that the locus of language change is in child language acquisition, in particular the process of inferring a grammar that is consistent with the sentences that have been heard [2–5]. Where these sentences do not fully specify a grammar, a child can infer a different grammar from its parents. If enough children infer a different grammar, then the language changes as the generations succeed each other. Variations on this basic idea exist, for example, where a child may have multiple grammars representing old and new linguistic variants, with the relative weighting of the two grammars shifting across generations [4]. A competing account is the usage-based theory [6–9], where linguistic innovation occurs at any point in a speaker’s lifespan, and speakers vary the frequencies that they use different structures incrementally across the lifespan [10–13].

One reason that this question has not been resolved during the century-long debate is that direct evidence of the origin of a change that develops into a new linguistic convention is generally lacking. Research in child language acquisition has demonstrated that children are very good at acquiring and conforming to the conventions of the speech community. In fact, the primary research question in child language acquisition is how children are so successful in mastering not only general rules of language but also the many exceptions and irregularities in adult language conventions [14]. Child-based approaches argue that children find the patterns rapidly on the basis of specific innate language structures, while usage-based approaches argue that child language acquisition is incremental and general patterns are expanded gradually [15]. The fate of any innovations that are produced in the acquisition phase tends not to be investigated in this line of research. Meanwhile, sociolinguistic research on variation and change begins with a situation in which the novel variant has already been produced, and in fact the novel variant is already changing in frequency on the way to becoming a new linguistic convention. It is virtually impossible to capture the innovation as it happens; linguists are always analysing situations in which the new variant is already present.

Hence linguists have tended to rely on indirect evidence that would shed light on the role of the individual in language change. For example, it has been observed that the sound changes that are produced by children—innovations, or “errors” from the perspective of adult grammar—are not the same as the sound changes that have been documented in language history [16–21]. However, the innovative variation produced spontaneously by adults in both sound and grammar *is* of the same type that has been documented in language history [22, 23]. These observations support the usage-based theory over the child-based theory. Also, while children are extremely good at acquiring the linguistic conventions of adults, by late adolescence they develop into the leaders propagating a novel variant through the speech community, which suggests that language change does not originate in childhood [10, 13, 24, 25].

Here we take a novel approach to addressing the question of the locus of language change in the individual: we quantify and compare the plausibility of different theories of individual behaviour in producing population-level language changes and the resultant worldwide diversity of language traits. We achieve this by introducing a mathematical model that allows us to test a variety of hypotheses about how individuals ultimately bring about language change at the population level. The model is applied to diachronic and crosslinguistic data of one common type of language change, the grammatical evolution of definite and indefinite articles, such as English *the* and *a* respectively. The evolution of articles can be analysed as a cycle of states in which a language without an article may develop an article which may then disappear, allowing a simple unidirectional model of innovation and propagation of a change in a finite set of states. We draw on data of attested changes in definite and indefinite articles for 52 languages, and on the cross-linguistic distribution of article states (620 languages for definite articles, 534 languages for indefinite articles; see below for further details).

Our model allows us to access a very wide range of different individual-level processes of language learning and use which appear in different combinations, whilst remaining amenable to mathematical analysis with methods from population genetics [26]. Specifically, we can estimate the likelihood of our set of empirical language changes at the population scale, given a certain set of assumptions on the behaviour at the individual level. This then means we can determine the regions within this model space that have the strongest empirical support. As we will show below, we find that explanations of language change that appeal exclusively to childhood language learning receive considerably less support than those that allow incremental change across the lifespan. Our analysis further suggests that the complex structure of social networks—in which the degree of influence that different speakers may have over others is highly variable—may play an important role in the diffusion of linguistic innovations.

## Data and methods

**Fig 1:**
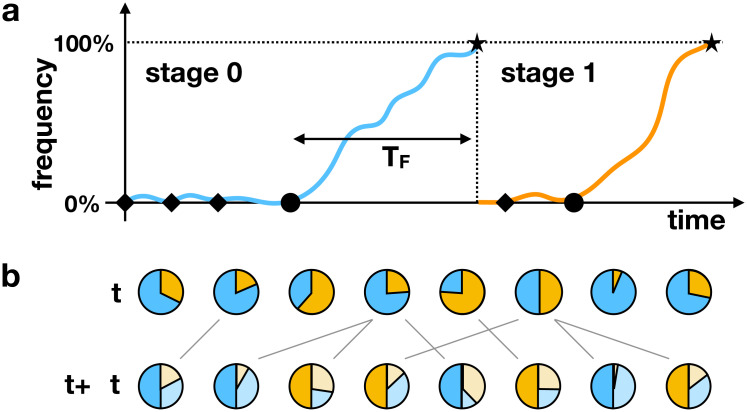
(a) Origin-fixation model at the population scale, showing a transition between two stages of a grammaticalisation cycle (set out in Table 1). Innovations are repeatedly introduced to the population; most fail (diamonds), but some successfully originate a change that propagates and goes to fixation (circles). The fixation time *T*_*F*_ is a random variable (see text). (b) Underlying individual-based (Wright-Fisher) model. Individuals are characterised by the frequency with which they use the innovation (orange portion of pie charts). In the case shown, individuals update their innovation frequencies by retaining a fraction 1 − *ϵ* their existing value, and acquiring the remaining fraction *ϵ* through exposure to one other member of the speech community. In the figure, $\epsilon =\frac{1}{2}$ for illustrative purposes. The two levels of description are connected by averaging over the individual speaker-level innovation frequencies in the Wright-Fisher model to obtain the population-level frequency plotted for the origin-fixation model.

In this section we first set out empirical properties of changes in articles that guide us towards a statistical model of language change over historical time at the population scale. The basic picture, illustrated in Fig 1a, is one in which the population is initially at some stage of the cycle, for example, the situation where there is no definite article (stage 0). As a consequence of individual speaker innovations, an article is occasionally introduced into the population by recruiting a pre-existing word for the article function. This is indicated by diamonds in the figure. In later stages, different linguistic processes lead to a divergence in form, reduction of that form to an affix and the loss of the form. Eventually, one of the innovations propagates so that its *frequency*, defined as the proportion of relevant contexts in which the innovation is used, rises to 100%. Once this occurs, the next stage of the cycle has been reached and the process begins afresh. Following [26], we refer to this population-scale model as an *origin-fixation model*: the introduction of an innovation that successfully propagates (denoted by a circle in the figure) is referred to as *origination*, and the point at which it reaches a frequency of 100% is called *fixation*.

This population-scale process is the product of interactions between individual speakers in the population, that is, acquisition or use, or a combination of the two. These interactions are illustrated schematically in Fig 1b and will be discussed in detail in the second part of this section. The individual-based model is very similar to the *Wright-Fisher model* in population genetics (see e.g. [27]), and we refer to it as such. In this model, each speaker is characterised by the frequency with which they use an innovation in the relevant linguistic context. The Wright-Fisher and origin-fixation models are connected by averaging over the individual frequencies to obtain the corresponding frequency at the population level. This then provides a quantitative model for language change over historical timescales that is grounded in individual speaker interactions.

### Language change at the population level

#### Empirical properties

We draw on two sources of data to characterise language change at the population level: (i) a survey of documented instances of historical language change (detailed in S1 Appendix); and (ii) the typological distribution of the current stage in the cycle across the world’s languages (as recorded in the World Atlas of Language Structures, WALS [28]). As stated in the Introduction, we focus on definite and indefinite articles for this analysis. There are a number of reasons for this. First, the evolution of articles predominantly follows a single cycle of grammaticalisation. Definite articles are predominantly derived from demonstratives such as *that* [29], and indefinite articles are predominantly derived from the numeral *one* [30]. Both articles proceed to being affixed and then disappear. Second, articles are unstable: several find articles to rank among the least stable of a large set of features [31–33]. This means that our historical survey includes many documented instances of multiple stages in the article grammaticalisation cycle, which in turn leads to a more sensitive likelihood-based analysis than is possible when changes are rare. Finally, this instability implies that the current distribution of stages in the cycle across languages is likely to be close to the stationary distribution, which simplifies the analysis. Although articles are at one end of the stability spectrum, we expect that similar results to those reported below would be found for more stable features: we return to this point in the Discussion.

We divide the stages of the cycle following the classification of WALS Features 37A and 38A [28]: (0) no explicit article; (1) use of *that* and *one* for definite and indefinite article meaning respectively; (2) use of a distinct word usually derived from *that* or *one* for the article; and (3) use of an affix. WALS provides the current crosslinguistic distribution of these four stages for definite and indefinite articles (see Table 1). One can also look at the joint distribution of the two features to establish whether they are correlated. A *χ*^2^ test on the contingency table indicates that the features are unlikely to be independent (*p* < 10^−6^; although the conditions for the validity of the *χ*^2^ test do not strictly apply, this level of significance was confirmed by a Monte Carlo sampling procedure).

**Table 1:** Typological distribution of definite and indefinite articles.

	Definite		Indefinite	
State	Description	Number	Description	Number
0	No article	243	No article	296
1	Same as *that*	69	Same as *one*	112
2	Distinct word	216	Distinct word	102
3	Affix	92	Affix	24

The number of languages in each state is taken from [28].

**Fig 2:**
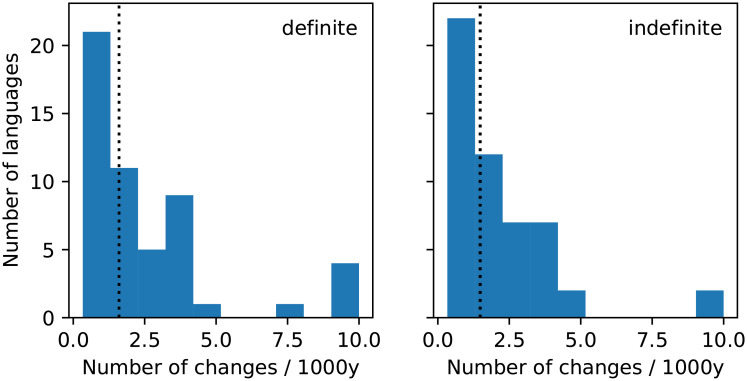
Distribution of the number of changes in the definite (left) and indefinite (right) article per 1000 years over the empirical dataset of 52 languages. The vertical dotted line indicates the median of the distribution.

We collected data on the documented history of articles in 52 languages from multiple sources (see S1 Appendix), and divided their history into the same four stages. Importantly, at any given point in time, one of these conventions typically dominates; over time the dominant convention changes to the next in the sequence 0–3 above, before returning to stage 0 via loss of the article. In our analysis of the 52 languages, we find only a single instance of a stage of the cycle that was skipped. For each article and language, we can estimate the rate of change as $\frac{m+1}{t}$, where *m* is the number of changes observed and *t* is the observation period. (Technically, this is the mean of the posterior distribution over rates when the prior is uniform and the changes assumed to occur as a Poisson process.) We plot the distribution of these rates for each article in Fig 2. This shows that the median rate of change is roughly once every 1000 years and that the distribution is somewhat skewed towards slower rates of change. Our survey further suggests that the time taken for a change to propagate is somewhat shorter than this, perhaps of the order of 100 years. We further find that, for any given language, the number of changes in one article is not independent of the other (*χ*^2^ test *p* = 0.00058; Monte Carlo estimate *p* = 0.0026). In the following we present results for the two articles separately, as combining probabilities from the two analyses is not justified when measurements are correlated.

#### Origin-fixation model

We use the historical properties of article grammaticalisation cycles, set out above, to flesh out our statistical model of the process at the population scale. Recall from Fig 1a the picture of an initial state in which all speakers are at a given stage of the cycle (say, stage 0), and as speakers interact, instances of the next stage are repeatedly introduced. In a child-based model [2, 4, 5], the next convention is introduced by children in the acquisition process. In the usage-based model, by contrast, the next convention is introduced in language use by speakers of any age [7, 9, 22].

Under whatever mechanism one has in mind, only some of the individual innovations are replicated sufficiently often that they become used by the entire population, reaching the frequency of 100% that defines the state of fixation and therewith the onset of the next stage of the cycle [22, 23, 34].

We assume that the rate at which speakers introduce a specific innovation (e.g., introducing a particular form for an article) in individual instances of acquisition or use is constant over time, as is the probability that this innovation then propagates and reaches fixation. This means that at any given stage in the cycle, origination events occur at a constant rate. In mathematical terms, origination is a Poisson process with rate *ω*_*i*_ when the population is in stage *i* of the cycle (and so the innovations correspond to stage *i* + 1).

Specifically, we take $\omega_{i}=\frac{\bar{\omega }}{4f_{i}}$, where *f*_*i*_ is the fraction of languages currently at stage *i* in the cycle (Table 1). This choice ensures, for any value of the parameter $\bar{\omega }$, that the stationary distribution of the origin-fixation is one in which the probability of being at stage *i* of the cycle is *f*_*i*_, and consequently matches the WALS distribution (although our conclusions do not depend on this being the case). By including the factor 4 (i.e., the number of stages in the cycle) $\bar{\omega }$ can be interpreted as a mean origination rate obtained by averaging over one complete cycle. In general we will treat this rate as a free parameter (see Results, below).

Once the originating innovation has entered the population, it takes a time *T*_*F*_, called the *fixation time* to become adopted as the convention by all speakers in the population. In origin-fixation models applied to the invasion of mutant genes in a biological population [26, 35], the origination process is generally much slower than the fixation process, and *T*_*F*_ is typically set to zero. This is not appropriate in the application to language change: the historical survey above suggests that *T*_*F*_ is only one order of magnitude smaller than the time between the origination of a change. Moreover, *T*_*F*_ is unlikely to be exactly the same for each change, due to the unpredictability of human interactions and individual speech acts.

**Fig 3:**
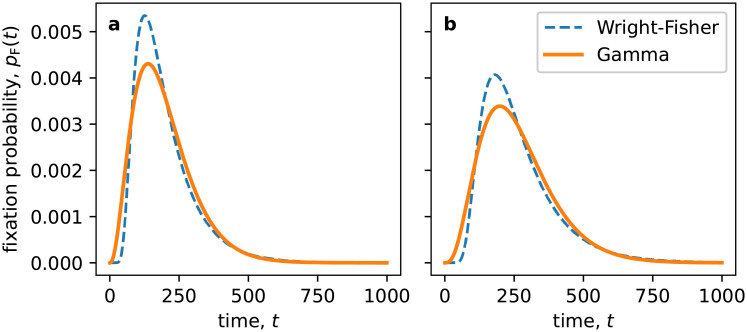
Approximation of the fixation time distribution obtained numerically for the Wright-Fisher model (dashed line) with a Gamma distribution given by Eq 2. In (a) the Wright-Fisher model has *N* = 100 individuals and no selection. In (b) *N* = 150 and *s* = 0.01.

We account for this unpredictability by drawing each fixation time *T*_*F*_ from a probability distribution. The fixation time distribution can be calculated for certain individual-based models, such as the Wright-Fisher model set out below [27, 36]. However, the mathematical form is too complicated to be of practical use, so we approximate it by the simpler Gamma distribution. This distribution is a natural choice for a quantity that is required to be positive (like a fixation time), and whose mean and variance can be controlled independently. In fact, we will arrive at the population-scale model by setting these two quantities equal to those that derive from an underlying individual-based model. Fig 3 shows the Gamma-distribution approximation to the fixation time distribution obtained numerically for the Wright-Fisher model with and without a selection bias. Although the Gamma distribution does not fit perfectly, it captures the location and width of the peak well, and is preferable to simply assuming that *T*_*F*_ is zero.

We now provide a formal mathematical definition of the origin-fixation model that is equivalent to the verbal description above. Starting from stage *i* of the cycle, a time *T*_*O*,*i*_ at which a change to the next stage in the cycle is originated is drawn from the exponential distribution

$$\begin{array}{c} P_{O,i}\left(T_{O,i}\right)=\omega_{i}{\text{e}}^{-\omega_{i}T_{O,i}} \end{array}$$ (1)

as is appropriate for a Poisson process. Then, the time *T*_*F*_ from origination to fixation is drawn from the Gamma distribution

$$\begin{array}{c} P_{F}\left(T_{F}\right)=\frac{\beta^{\alpha }}{\Gamma (\alpha )}T_{F}^{\alpha -1}{\text{e}}^{-\beta T_{F}}\;\text{where}\;\alpha =\frac{{\bar{T_{F}}}^{2}}{\sigma_{F}^{2}}\;\text{and}\;\beta =\frac{\bar{T_{F}}}{\sigma_{F}^{2}}\;. \end{array}$$ (2)

At this point, stage *i* + 1 is entered, and origination of a change to stage *i* + 2 can begin (by sampling a Poisson process and Gamma-distributed fixation time, as above).

The crucial point is that once these distributions are specified, one can compute the likelihood of the observed changes in our historical survey for any desired combination of parameters *ω*_*i*_, $\bar{T_{F}}$ and $\sigma_{F}^{2}$. Specifically, we ask for the probability that a language in stage *i* at the beginning of the observation period reaches stage *j* by the end of that period. The set of periods, changes, and procedure for calculating the likelihood are detailed in S1 Appendix. In the likelihood calculation, each language is treated as independent of the others: we do however consider a mother and its daughters after a split as separate languages, so that changes in the mother language are not included multiple times in the sample. It is important to note that the origin-fixation parameters are *not* arbitrary, but depend on the underlying behaviour of individuals. A specific choice of individual-based model will lead to specific values of the parameters *ω*_*i*_, $\bar{T_{F}}$ and $\sigma_{F}^{2}$, as we establish below.

### Language change at the individual level

#### Wright-Fisher model

We now set out a model of language behaviour at the individual level which allows us to determine parameter values for the origin-fixation model in regimes of interest. We start with the fact that all theories of language learning and use involve the linguistic behaviour of one individual in the population being adopted (in some way) by another. Looking backwards in time, one can construct a ‘genealogy’ that shows who acquired linguistic behaviour from whom, analogously to the inheritance of genetic material under biological reproduction. It is well understood in population genetics that many superficially different individual-based models of inheritance generate a common distribution of genealogies [37]. Therefore, one obtains a generic and robust description of an evolutionary process by selecting a specific individual-based model that is adapted to the context at hand. Here we construct a model of the Wright-Fisher type [27] that allows us to manipulate key properties of the individual speaker, such as how often they can change their behaviour (though learning or use, as appropriate), whether biases towards or against the innovation are operating, and which other members of the speech community they interact with.

The basic structure of this model is shown in Fig 1b. Each circle in the figure represents an individual’s linguistic behaviour at a given point in time. Each individual uses the existing convention (stage 0 in the figure) some fraction of the time, and the incoming innovation (stage 1) the remaining fraction of the time. As in the origin-fixation model, we assume that at most two linguistic variants are widely used at any given time. A variable *x*_*n*_ specifies the relative frequency (in the range 0 to 1 inclusive) that speaker *n* uses the innovation. For example, the left-most speaker in the figure is using the innovation in around $x_{1}=\frac{1}{3}$ of the relevant contexts at time *t*. In this work, we take *x*_*n*_ to be an average over occurrences of a particular form of the article in a general Noun Phrase construction that expresses (in)definiteness of the referent of the Noun Phrase. The forms are: no article; article identical to a source form (demonstrative for definite article, the numeral ‘one’ for indefinite article); article distinct from source form; and article attached to noun. Although this general construction may be made up of more specific subtypes of Noun Phrase constructions, there is reason to believe that a regular trajectory of change emerges from the aggregation of occurrences over subtypes [38].

In the traditional Wright-Fisher model, *x*_*n*_ takes only the extremal values 0 or 1. In a linguistic context, this corresponds to classic child-based models [2, 3, 5] in which a speaker’s grammar is specified in terms of binary parameters. Other models allow for intermediate values of *x*_*n*_: these include variational learning [4] and usage-based [15] models.

The innovation frequencies *x*_*n*_ are updated at a rate *R* for each of the *N* speakers in the population. We define the update rule in a way that includes the child- and usage-based models as special cases. What these have in common is that, in an interaction, each individual is exposed to the behaviour of one other speaker in the population. Each then replaces a fraction *ϵ* of their stored linguistic experience with a record of the variant that was perceived in this interaction. That is, $x_{n}^{\prime }=\left(1-\epsilon \right)x_{n}+\epsilon \tau$, where $x_{n}^{\prime }$ is the updated innovation frequency, and *τ* = 1 if the innovation was perceived in the interaction, and *τ* = 0 otherwise. Fig 1b illustrates this update for the case $\epsilon =\frac{1}{2}$.

The child-based model is obtained when *ϵ* = 1. The update then corresponds to a child being exposed to the behaviour of a parent, applying some learning rule to determine if the grammar of the language corresponds to the convention or the innovation, and setting *x* = 0 or 1 accordingly. Importantly, the learning rule can allow the child to infer a grammar that is different from that of the parent: cue-based learning [39] is one mechanism that allows for this. A general model for such mechanisms can be obtained by introducing a probability *η*_*i*_ that, given a behaviour that is consistent with the parent holding grammar *i* in the cycle, the child nevertheless adopts grammar *i* + 1 (for example, because the sentences produced by the parent are more consistent with the next stage of the grammaticalisation cycle). In the child-based model, the appropriate choice for the update rate *R* would be once per generation. Under these conditions, the timescale of the cultural evolutionary process of language change is necessarily tied to that of biological evolution (although the two processes differ in other respects, for example, the number and identity of parents).

By contrast, the usage-based model allows for the cultural evolutionary dynamics to proceed more quickly than their biological counterparts, as individuals interact many times in the course of a generation. However, the impact of each interaction is likely to be smaller, implying that the parameter *ϵ* that quantifies this impact should be small. Fig 1b illustrates the case of $\epsilon =\frac{1}{2}$, in which after the update (time *t* + Δ*t*), half of the usage frequency derives from their behaviour before the interaction (light shading in the figure), and the other half (dark shading) corresponds to whether a conventional or innovative utterance was perceived in an interaction with the speaker shown by the connecting line. As in the child-based model, there is a small probability *η*_*i*_ that a conventional behaviour is perceived as an innovation. This can represent a variety of processes that might apply in single instances of use, e.g., auditory and articulatory constraints [40, 41] or cognitive biases [41–43], along with indeterminacy in inferring a phonological form [22, 34] or meaning [23, 44], that may favour one construction over another (see e.g. [7] for an extended discussion of innovation in language change).

To complete the description of the Wright-Fisher model, we need to specify how the *interlocutor*—the speaker who provides the linguistic data to the learner (or listener)—is chosen. There are two components to this: (i) a social network structure; and (ii) a possible biasing of interlocutors based on their linguistic behaviour. We describe these in turn.

**Fig 4:**
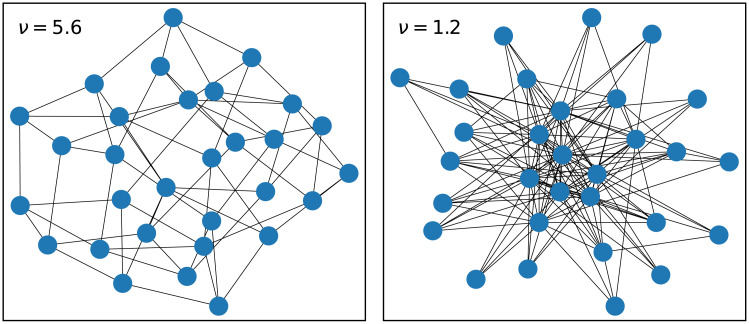
Instances of random networks with different degree exponents *ν*. The case *ν* > 2 (left) corresponds to a homogeneous network in which individuals all have a similar number of neighbours. The case *ν* < 2 (right) is heterogeneous: the central individuals are well-connected whilst the peripheral individuals are not.

The social network is set up so that speaker *i* has *z*_*i*_ immediate neighbours, with *z*_*i*_ drawn from a *degree distribution* *p*_*z*_. Thus different individuals can have different numbers of neighbours. In the absence of the bias, each neighbour is chosen as an interlocutor with equal probability in an interaction. A generic model for social networks is the power-law degree distribution *p*_*z*_ ∝ *z*^−(1 + *ν*)^ in which the exponent *ν* controls the heterogeneity of the network. Values of *ν* > 2 are regarded as homogeneous, in the sense that innovations spread in the population in the same way as on a network in which all speakers have the same number of neighbours (even though there is variation). When *ν* < 2, the networks become increasingly heterogeneous as *ν* is decreased: these feature a small number of highly-connected individuals and a large number of relatively isolated individuals. Evolutionary dynamics tend to run faster on heterogeneous networks [45–47], and there is some evidence that human social networks are heterogeneous (1.1 < *ν* < 1.3, [48–50]). Fig 4 illustrates the distinction between homogeneous and heterogeneous random networks.

The interlocutor bias is implemented by choosing a neighbour *m* with a probability proportional to 1 + *sx*_*m*_ instead of uniformly. The *selection strength* *s* serves to favour (if *s* > 0) or disfavour (if *s* < 0) the innovation, which may originate in one of a number of processes. For example, in the variational learning framework [4], there is a systematic bias towards a grammar that parses a larger number of sentences. In a sociolinguistic setting, association between a linguistic variant and a socially prestigious group may lead to a bias towards (or against) that variant [10, 51]. The case *s* = 0 describes a neutral model for language change, which has been discussed in the context of new-dialect formation [52, 53].

We emphasise that a large number of models for language learning and use that have been discussed in the literature fall into the Wright-Fisher class, even though they may differ in detail and may not be presented as such. A non-exhaustive list includes those that appeal to cue-based learning [39], Bayesian learning from one or more teachers [54–56], variational learning [4] and usage-based models [57]. Moreover, the Wright-Fisher model has been used as a phenomenological model for changes in word frequencies [58–60].

We conclude this section with a formal mathematical specification of the Wright-Fisher model. The distribution *P*(*x*, *t*) of the innovation frequency, *x*, at the population level, at a time *t* after it is originated, is generally well-described by the forward Kolmogorov equation

$$\begin{array}{c} T_{M}\dot{P}\left(x,t\right)=-s{\left[x\left(1-x\right)P\left(x,t\right)\right]}^{\prime }+\frac{1}{2N_{e}}{\left[x\left(1-x\right)P\left(x,t\right)\right]}^{\prime \prime } \end{array}$$ (3)

in which a dot and prime denote derivatives with respect to *t* and *x*, respectively [27, 61]. The parameters *T*_*M*_, *s* and *N*_*e*_ correspond to a memory lifetime, an innovation bias and an effective population size, respectively. We emphasise that this equation applies between successive origination events, and describes the process by which the innovation propagates (rises to *x* = 1) or fails (falls to *x* = 0). Therefore the origination rate does not appear in this equation. However, it does enter into a correction factor, set out in S1 Appendix, that accounts for the possibility that a second origination occurs before either of these endpoints is reached.

The main difference between models within the Wright-Fisher class is how *T*_*M*_, *s* and *N*_*e*_ relate to the parameters that apply to a specific model. In the present case, which has the set of parameters specified in Table 2, we have *T*_*M*_ = 1/(*Rϵ*), *s* is as specified above and $N_{e}=N\left({\bar{z}}^{2}/\bar{z^{2}}\right)/\epsilon$ in which *z* is the number of neighbours a speaker has on the social network, and the overline denotes an average over speakers [45–47].

**Table 2:** Parameters in the individual-based Wright-Fisher and population-level origin-fixation models.

Wright-Fisher model		Origin-Fixation model	
Symbol	Meaning	Symbol	Meaning
*N*	speech community size	*ω*_*i*_	origination rate
*ν*	social network heterogeneity	$\bar{T_{F}}$	mean fixation time
*R*	interaction rate	$\sigma_{F}^{2}$	variance in fixation time
*ϵ*	interaction impact		
*η*_*i*_	innovation rate		
*s*	selection strength		

The parameters in the Origin-Fixation model that characterise the dynamics at the population scale can all be expressed in terms of those relating to the behaviour of individuals (see Data and Methods).

In S1 Appendix we demonstrate that Eq 3 applies more generally than to the specific agent-based model set out here, and furthermore that the quantities *T*_*M*_, *s* and *N*_*e*_ have a similar interpretation. This is achieved by considering a model that has many additional features—for example, ongoing birth and death of speakers, changes in social network structure and variation in interaction rates between speakers and over time—and showing that the changes in the innovation frequency *x* over short time intervals are the same as those described by Eq 3. Therefore the results we present below do not rely on this model being an accurate representation of language learning and use.

#### Connection to origin-fixation model

We connect the individual to the population scale by determining how the parameters in the origin-fixation model (also specified in Table 2) relate to those in the Wright-Fisher model. The origination rates *ω*_*i*_ are given by the formula *ω*_*i*_ = *NRη*_*i*_ *Q*(*ϵ*/*N*), where *N* is the number of speakers in the speech community, *η*_*i*_ is the individual innovation rate per interaction, *R* is the interaction rate and *Q*(*x*_0_) is the probability that an innovation goes to fixation starting from some frequency *x*_0_. In the Wright-Fisher model, this initial frequency is *x*_0_ = *ϵ*/*N*, because exactly one speaker uses the innovation with probability *ϵ*. We then have

$$\begin{array}{c} Q(\frac{\epsilon }{N})=\frac{1-{\text{e}}^{-2N_{e}s\epsilon /N}}{1-{\text{e}}^{-2N_{e}s}}\;. \end{array}$$ (4)

This result is obtained by solving the backward equation that corresponds to Eq 3 (see [27, 36] and S1 Appendix). We see that the effective population size, *N*_*e*_ (which depends on the actual population size *N*, the update fraction *ϵ* and the social network structure) plays an important part in determining the probability that an innovation propagates. It also determines how quickly an innovation may reach fixation. Numerical methods, described in S1 Appendix with the code available at [62], are used to determine exactly how the mean and the variance in the fixation time, $\bar{T_{F}}$ and $\sigma_{F}^{2}$, in the origin-fixation model depend on the Wright-Fisher model parameters. Here we note that the characteristic timescale is of order *T*_*M*_ *N*_*e*_ when the bias *s* is small, and of order *T*_*M*_ ln(*N*_*e*_) when it is large, which turns out to have important consequences for the plausibility of the historical data for specific models of language learning and use in our analysis below.

In summary, then, our basic approach is to use the origin-fixation model to determine the likelihood of an observed set of historical language changes. The parameters in this model are obtained from an underlying Wright-Fisher model, so that we may understand—for example—which learning rates, biases and social network structure are more or less well supported by the historical data. As we have argued, our findings do not depend on the detailed structure of the Wright-Fisher model. The crucial component is that a speaker’s behaviour can be represented by an innovation frequency *x*, and that this is affected by learning from or using language with other members of the speech community over time.

## Results

We now compare the likelihood of the empirically attested set of language changes (detailed in S1 Appendix) under different assumptions on the underlying behaviour of individuals in the respective populations. An appropriate measure for likelihood comparison is the Akaike Information Criterion, corrected for small sample sizes (AIC_*c*_, [63]), as the models we consider have different structures. It is defined as

$$\begin{array}{c} {\text{AIC}}_{c}=2k-2\ln\left(L\right)+\frac{2k(k+1)}{n-k-1} \end{array}$$ (5)

where *k* is the number of free parameters in the model, *n* is the number of observations and L is the likelihood of those *n* observations, as determined from the origin-fixation model. An observation is the sequence of transitions between different stages of a grammaticalisation cycle over a specified historical time period for a given language, as tabulated in S1 Appendix. The number of observations is therefore the number of languages in the sample (52 for both articles).

The difference in the AIC_*c*_ value between two models, denoted ΔAIC_*c*_, gives a measure of how much the model with the lower AIC_*c*_ score is preferred over the other. Models with more free parameters (higher *k*) can be dispreferred even when the data likelihood increases as a result of increasing parameters. For nested models, this increase is inevitable, but for models with different structures, AIC_*c*_ remains valid as it is based on general information theoretic principles [63]. Given two candidate models and a sufficiently large number of observations, ${\text{e}}^{\Delta {\text{AIC}}_{\text{c}}/2}$ provides an estimate of the probability that the model with the higher AIC_*c*_ better describes the data than that with the lower value. There is some freedom to choose the value of ΔAIC_*c*_ at which one discards the inferior model. In this work we take a value of around 10 (corresponding to a likelihood ratio of around 150) as indicative of the model with the higher AIC_*c*_ becoming too implausible to consider further. However since there is some flexibility in this regard, we will generally show the dependence of ΔAIC_*c*_ on model parameters, so one can gauge the scale of the likelihood differences between models. It is important to note that such model comparisons do not in themselves validate the superior model: for this one needs to consider goodness-of-fit measures as well [63].

We begin by establishing a baseline against which different individual-level mechanisms of language change will be compared. In this baseline model, language changes occur at the population level as a Poisson process. We emphasise from the outset that this is not an individual-based model of language change: changes in the population occur autonomously without reference to individual speakers. Nevertheless this model helps to illustrate our statistical approach and, as we discuss below, it also provides valuable insights into *why* particular individual-based mechanisms are found to provide more or less plausible explanations of historical language changes at the population level.

### Poisson baseline

In the baseline model, we assume that a change from stage *i* to stage *i* + 1 of the cycle occurs as a Poisson process at a constant rate $\omega_{i}=\bar{\omega }/\left(4f_{i}\right)$ in each population, where *f*_*i*_ is the fraction of the world’s languages that is currently at stage *i* of the cycle (Table 1). This factor of *f*_*i*_ ensures that the stationary distribution in the baseline model matches the contemporary WALS distribution. This model is equivalent to the origin-fixation model of Fig 1a, with instantaneous fixation (*T*_*F*_ = 0). This model has one free parameter, the mean rate of language change, $\bar{\omega }$, which is estimated by maximising the likelihood of the data.

The maximum likelihood value of $\bar{\omega }$, the corresponding AIC_*c*_, a classical *p*-value and two goodness-of-fit statistics are presented in Table 3. The *p*-value is the probability, within the model, of all possible transitions between stages of the relevant grammaticalisation cycle over the relevant historical period for each language whose likelihood is lower than the transitions that actually occurred. This *p*-value can be interpreted in the usual way, with a low *p*-value indicating a likely departure from the model assumptions.

**Table 3:** Fit of a Poisson process to article grammaticalisation histories.

	Definite	Indefinite
$\bar{\omega }(\times 10^{-4}\;{\text{yr}}^{-1})$	6.05	5.67
AIC_*c*_	128	93.6
*p*	0.0097	0.16
Overdispersion (number of changes)	2.7	1.1
Overdispersion (at least one change)	1.1	1.0

$\bar{\omega }$ is the maximum likelihood rate of change and AIC_*c*_ the corrected Akaike information criterion. *p* is the cumulative probability of events less likely than the observation. Overdispersion measures goodness of fit, with values closer to 1 indicating a better fit. *p* and overdispersion are estimated from 10^6^ Monte Carlo simulations of the process.

By itself, an AIC_*c*_ score (or differences between them) does not furnish any information about how well a particular model fits the data. To gain an insight into goodness-of-fit, we consider the *overdispersion* of two random variables *X* (specified below) which quantifies the extent to which observed deviations of *X* from their mean values $\bar{X}$ within the model are consistent with the expected deviations. For a given observation, the overdispersion is defined as $O_{X}={\left(X-\bar{X}\right)}^{2}/\text{Var}(\text{X})$, that is, the ratio of the observed square deviation to its expected value. If the overdispersion is close to 1, the deviations are as expected, and we conclude that the distribution of *X* is well-predicted [63]. For a given language, the two quantities *X* are: (i) the total number of language changes in the historical period; and (ii) a binary variable that equals 1 if at least one change occurred, or 0 otherwise. We average over all languages in the sample to obtain the single measure that is presented in Table 3.

The low overdispersion scores suggest that this baseline model provides a good description of changes in the indefinite article, whilst it performs less well for the definite article. A likely source of this difference is the larger number of languages whose definite article changes rapidly compared to the indefinite article, as can be seen from Fig 2. It is further possible that assumptions made about the data (for example, that the distribution of articles is stationary, that changes in different languages are independent, or, indeed, that the fixation time can be idealised to zero) do not strictly hold. We also remark that the second overdispersion measure is less sensitive than the first: however, it turns out that this is easier to calculate for individual-based models, and we will take a large deviation of this measure from 1 as providing a strong indication of a poor fit to the data.

It is remarkable that this simple model seems to provide a reasonably good fit to the data, particularly in view of an ongoing discussion about the role of population size in language structure and change [64–67] (a point we return to in the Discussion). The Poisson model explicitly assumes that the phenomenological rate of change $\bar{\omega }$ is constant across all populations, and that each language change is able to propagate rapidly from origination to fixation. These observations suggest that we should expect to find more plausible accounts of historical language change in individual-based models whose emergent population-level dynamics share these properties.

### Child-based models of language change

We now examine the constraints on the population-level dynamics of language change that arise from assuming that language change occurs primarily through the process of childhood language acquisition (e.g., [2, 4, 39, 54, 56, 68, 69]). As noted above, such theories imply that the rate, *R*, at which a grammar can be updated is once per human generation, which we take to be once every 25 years (i.e, *R* = 0.04yr^−1^). In the case where learning causes children converge on a single grammar (i.e., categorical use of one of the four article variants), we take *ϵ* = 1. In the case of variational learners (e.g. [4]), speakers can entertain mixtures of grammars: this can be realised with *ϵ* < 1. We consider the categorical case first.

The literature on child-based theories rarely refers to population structure. We therefore begin by assuming that populations are homogeneous: that is, that each child learns from roughly the same number of (cultural) parents, and conversely, that each adult provides linguistic input to roughly the same number of (cultural) offspring. Under these conditions, the emergent origination rates and fixation times in each population depends on a core size that is equal to the population’s actual size (see Methods). It is therefore necessary for us to estimate the population (speech community) size for each language over the historical period for which empirical data exist. In S1 Appendix, we set out the procedure that we use to estimate the mean population size for each language over its recorded period of change. This is then used as the core population size for that language in our analysis.

**Fig 5:**
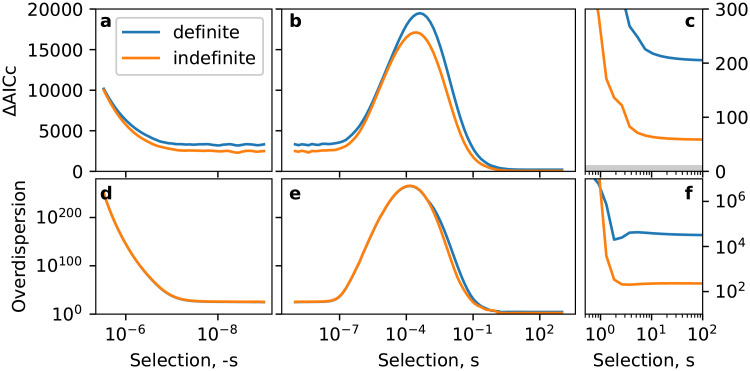
ΔAIC_*c*_ (panels a–c) and binary overdispersion (c–f) for negative (a and d) and positive (b, c, d and f) selection strength *s* within a child-based learning paradigm. The smallest values of both measures (which indicate better fits to the data) are obtained for strong positive selection (*s* > 1, highlighted in panels c and f which has a larger vertical scale). The ΔAIC_*c*_ values are far away from the shaded zone where ΔAIC_*c*_ ≤ 10 and the evidence in favour of the child-based model starts to become comparable with that of the baseline.

This leaves just two unconstrained parameters, the mean rate $\bar{\eta }$ at which innovations arise in individual instances of language learning (the “error” rate, in the child-based model), and the selective bias *s* in favour of the innovation. Our strategy is to choose the value of $\bar{\eta }$ that maximises the likelihood of the data set given all other parameter settings, and to plot ΔAIC_*c*_ with respect to the Poisson baseline model as a function of the selection strength *s* so that we can see where the support for the child-based model is strongest. Here, we treat the individual-based model as the candidate model, so ΔAIC_*c*_ = AIC_*c*_(candidate) − AIC_*c*_(baseline) is positive when the evidence supports the baseline model, and negative when the evidence supports the candidate model. The resulting plot is shown in Fig 5, along with a corresponding plot of the second of the two overdispersion measures considered for the Poisson baseline model.

We find that across the entire range of selection strengths *s*, support for the child-based model is very poor. The greatest plausibility (relative to the Poisson baseline) is obtained where ΔAIC_*c*_ is smallest: this happens in the limit of infinite selection strength. As can be seen from the rightmost panels of Fig 5, the values of ΔAIC_*c*_ in these regions are still rather large, reaching asymyptotes at 204 and 58.4 for definite and indefinite articles, respectively (both to 3 s.f.). This corresponds to the evidence in favour of the candidate model being 10^44^ (definite) and 10^13^ (indefinite) times smaller than the baseline.

However, this comparison with the Poisson baseline is not entirely fair, as this phenomenological population-level dynamics may not be accessible for any combination of parameters in the individual-based model. For this reason we must also check the goodness-of-fit via the overdispersion measure. Again we find anomalously large values, the asymptotic values being 31300 (definite) and 226 (indefinite), suggesting that the assumptions made about the underlying dynamics of language change are wildly inconsistent with the historical data. Throughout this investigation, we found that ΔAIC_*c*_ correlates strongly with goodness-of-fit, and so in the rest of this work we show only ΔAIC_*c*_, and investigate whether alternative assumptions on the individual-level behaviour are capable of delivering a much smaller ΔAIC_*c*_.

To focus this investigation, it is instructive to understand why the empirical data have such a low likelihood (and therewith high ΔAIC_*c*_) within the child-based model. As previously noted, the effective population size (which here, is the same as the actual population size) is of fundamental importance in population genetics models [27]. When the selection strength, *s*, is large, each individual innovation is likely to propagate, and the mean origination rate (at the population level) increases linearly with the population size. On the other hand, when the selection strength is small, the origination rate is roughly constant but fixation time *T*_*F*_ is proportional to the population size. Since the historical average population sizes in the empirical data set range across six orders of magnitude, then either the origination rate or the fixation time exhibits this wide variation in the child-based model. The fact that the Poisson baseline, which has no dependence on population size at all, apparently provides a much better fit, suggests that individual-based models in which origination rates and fixation times vary more weakly with population size than in the child-based model should be more favoured. Variants of the child-based model in which grammars are probabilistic [4] do not fall into this class: these have *ϵ* < 1, which implies a fixation time *N*/*ϵ*^2^ when *s* is small. That is, these models are more sensitive to population size than models that allow children to acquire only a single grammar.

### Usage-based models of language change

In a usage-based model, a speaker’s grammar may change across their lifespan [15], in principle in response to every utterance they hear (i.e., up to around 10^7^ times a year [70]). This has the potential to weaken the sensitivity to population size: if a large number of interactions between speakers is required for a change to propagate through the population, then the higher interaction frequency in the usage-based model gives the change a greater chance of going through on the attested historical timescales. However, this effect may be tempered by the fact that the change to each grammar is smaller in each interaction, which has the opposite effect.

**Fig 6:**
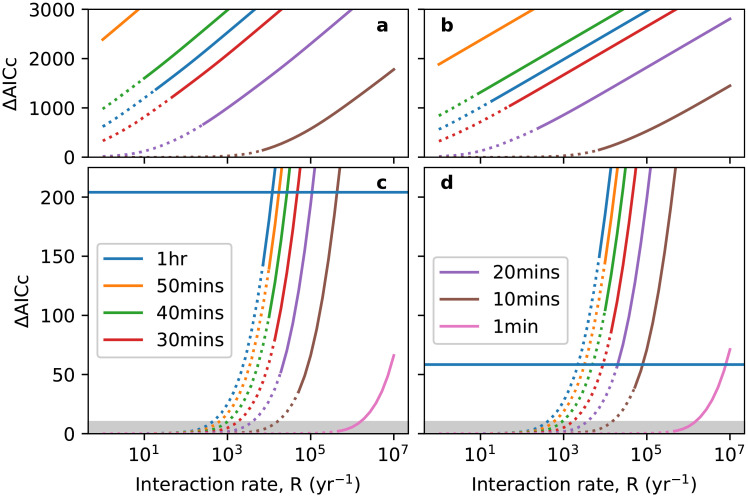
ΔAIC_*c*_ in the usage-based model as a function of interaction rate *R* for the definite (panels a and c) and indefinite (b and d) articles. Along each curve, the memory time *T*_*M*_ = 1/(*Rϵ*) is held constant. In panels a and b, *T*_*M*_ ranges from 25 years (top line) to 1 hour (bottom line). Panels c and d focus on the range of interest where greater plausibility than the child-based model is achieved: the horizontal lines correspond to the *s* = ∞ asymptotes in Fig 5. Dotted lines indicate where the usage-based model is unphysical (*ϵ* > 1) and the shaded grey region indicates where the fit starts to become comparable to the Poisson baseline (ΔAIC_*c*_ < 10).

To explore the interaction between an increased interaction rate *R*, and lower impact on the grammar *ϵ*, it is convenient to work with the memory time *T*_*M*_ = 1/(*Rϵ*), which is the expected lifetime of a single item of linguistic experience in the speaker’s mind. Considering again the case of homogeneous populations, we compare in Fig 6 the class of usage-based models with no selection (*s* = 0) over the reasonable range of *R* at fixed memory times *T*_*M*_ = 1/(*Rϵ*) against the baseline model. Note that the dotted parts of the curves correspond to an unphysical parameter value of *ϵ* > 1. From these ΔAIC_*c*_ plots, we see that our intuition that an increased interaction rate allows changes to go through more easily is correct. We achieve greater plausibility than the most plausible child-based model when memory times are short, specifically less than one hour. We note that we can approach the plausibility of the Poisson baseline if we allow *T*_*M*_ to be as short as one minute.

Although shorter memory times in the individual allow for a faster rate of change in the population, the basic property of fixation times being proportional to the population size is unaffected. This is why we find that individual memory times must be very short (perhaps unreasonably so, see Discussion) to improve on child-based models. Furthermore, there is stronger sensitivity to population size when selection is operating (*s* ≠ 0), which leads to lower plausibility gains with respect to the child-based model than in the neutral case (*s* = 0). This suggests that one needs to appeal beyond merely shorter memory times to explain the apparently weak effect of population size on article grammaticalisation cycles.

### Social network effects

Studies of the Wright-Fisher and related models on heterogeneous networks [45–47] show that these can weaken the effect of population size on characteristic timescales of change. As discussed in the Wright-Fisher model section, above, we model social networks as those with a power law distribution *P*(*z*)∼*z*^−(1+*ν*)^. We recall that the exponent *ν* controls the heterogeneity of the network, with lower values of *ν* corresponding to greater heterogeneity: see also Fig 4. On such networks, the mean fixation time is proportional to an *effective* population size *N*_*e*_ ∼ *N*^2−2/*ν*^ which is less than the actual size *N* if 1 < *ν* < 2 [45–47]. In the context of language change, we can think of *N*_*e*_ as measuring the size of a core population who exert much greater influence over the periphery than vice versa. Empirical studies of large networks (like friendship networks) provide some support for this power-law distribution with an exponent *ν* in the range 1.1 < *ν* < 1.3 [48–50].

**Fig 7:**
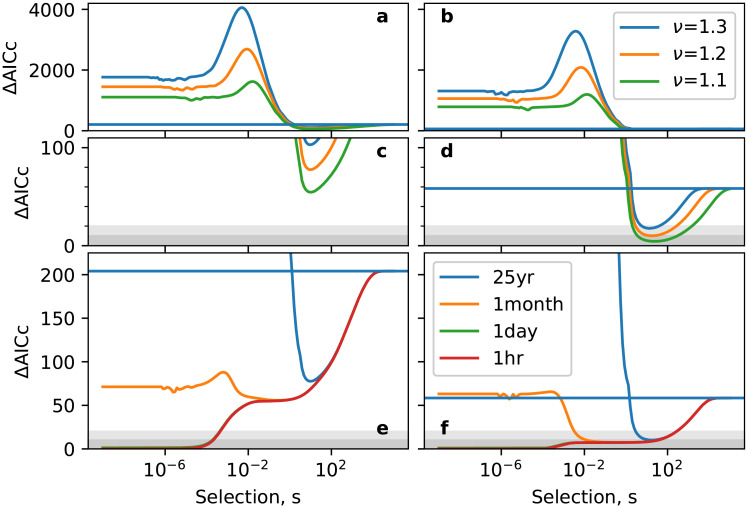
ΔAIC_*c*_ for models on heterogeneous social networks for the definite (panels a, c and e) and indefinite (b, d and f) articles as a function of selection strength *s*. Panels a–d show the effect of different degree exponents *ν* on the child-based model: panel c and d zoom in on ΔAIC_*c*_ ≤ 100, showing that plausibility is obtained only for the indefinite article over a limited range of *s* and *ν*. Panels e and f show the effect of memory lifetime at fixed *ν* = 1.2 and *ϵ* = 1. The horizontal line has the same meaning as in Fig 6. The dark and light shaded regions correspond to ΔAIC_*c*_ < 10 and ΔAIC_*c*_ < 20, respectively, which allows one to see the sensitivity to different evidence thresholds.

In Fig 7 we examine how the plausibility of both the child- and usage-based models investigated above changes when individual speakers in the model are arranged on complex network structures. This confirms our expectation that models in which timescales of change are less sensitive to population size receive greater support from the data. As previously, the usage-based model provides a more plausible description of language change than the child-based model; moreover, the range of selection strengths and memory times over which a fit comparable to that provided by the Poisson process is much larger than on homogeneous networks.

We see from Fig 7 that the most plausible models in the space under consideration are those in which selection is relatively weak. This is consistent with recent observations [58–60] that the dynamics of word frequencies appear to be subject to the evolutionary forces of both random drift and selection (i.e., neither is so strong that it dominates the other). Moreover, a number of studies (e.g., [46, 71, 72]) have indicated that heterogeneity tends to lower the barrier to invasion of an infection, mutation or innovation. This possibly points towards a picture whereby the different grammatical structures that are attested cross-linguistically are somewhat similar in their fitness, but may nevertheless replace one another over time in the systematic way that is observed historically due to the manner in which human societies are structured.

## Discussion

The aims of this work were twofold. First, we established how specific assumptions on the way in which individuals learn and use language translate to language change at the population scale. Second, we used historical data for the latter to identify which theories and mechanisms as to how individuals change the language of their speech community have greater empirical support.

Our main result is that if we impose the constraints that arise from assuming that childhood language learning is the driver of language change, there is no combination of the remaining free parameters that provides a good fit to the empirical data. The observed changes are many orders of magnitude more likely in regions of parameter space that correspond to other theories. The reason why the support for the child-based theory is so poor lies in a strong dependence of characteristic timescales at the population level on the underlying population size. If any selective bias in favour of the innovation is weak, the time taken for a change to propagate through a large speech community (the fixation time) is very much longer than the 100 years or so that is seen historically. If selection is strong, changes propagate quickly but then the rate at which successful changes are originated varies strongly with population size. The empirical data apparently show much less sensitivity to population size than the child-based theory implies.

In fact, throughout this work, we have found that the baseline model, which has no dependence on population size, fits the historical data well. One way to construe the baseline model is as changes originating once every 1000 years or so in every population, with changes then propagating rapidly through the population. This suggests that the mechanisms that have stronger empirical support are those that have these characteristics.

We acknowledge that our analysis is based on a single pair of features (the definite and indefinite articles) that are relatively unstable and are correlated. It is due to these correlations that we treated them separately (rather than combining them together into a single likelihood measure, which would assume independence). Nevertheless, comparison of the two articles is informative about how sensitive the analysis is to the details of which languages undergo a specific sequence of changes, as this does vary between the two articles. Overall, we find that it is the overall rate of language change combined with its weak sensitivity to population size that most strongly determines the plausibility of a given individual-based theory.

It is, however, possible that the dynamics of articles are unrepresentative of grammatical features more generally, and that our conclusions therefore do not generalise. We argue that this is unlikely. Regarding overall timescales of change, it is well established, by different analyses [31–33], that articles rank amongst the least stable of grammatical features and that others change more slowly. Basic word order lies at the opposite end of the spectrum, and the lifetime of given word orders have been estimated as ranging from 1000–100000 years [73]. That is, these most stable structures persist for a timescale that ranges from around the same order of magnitude as articles to two orders of magnitude longer. A quick way to estimate the plausibility of the child-based theory for basic word order from our findings for articles is to consider a generational turnover that is increased by two orders of magnitude (i.e., from 25 years to around 3 months). Here we find a plausible account is possible on sufficiently heterogeneous social networks (see Fig 7). This implies that the child-based theory could, at best, account for only the most stable grammatical structures, and does not offer a single explanation for language change that applies across the stability spectrum. The rate of population turnover imposes a fundamental minimum rate of language change which lies above that for unstable features in the child-based account, but potentially below in the usage-based account. Therefore the latter is capable of providing a common explanation for changes across the full stability spectrum.

It is harder to establish whether the weak sensitivity to population size is a feature of other grammatical changes. A detailed record of the history of each feature of interest across many languages is required for a conclusive assessment, data that is difficult to obtain (particularly for more stable features, where greater time depth is required to see a sufficiently large number of changes). However, a number of studies that have directly examined the relationship between population size and various aspects of language structure or change [64–67] have tended to conclude that where there is an effect, it is weak. For example, [67] reports rates of gain and loss that scale sublinearly with the population size, consistent with the behaviour of Wright-Fisher models on heterogeneous social networks. Moreover, the fact that different methods [31–33] of characterising the stability of a feature with a single metric are broadly consistent suggests that they do not vary significantly over space and time. Indeed, Wichmann and Holman [31] have argued that the notion of stability is intrinsic to a feature and does not vary geographically. Given these considerations, it seems reasonable to conclude that weak population-size dependence is a generic property of language change, and not peculiar to articles.

We have identified two individual-level mechanisms that may contribute towards such a weak effect of population size on the rate of grammatical change. The first of these is provided for by usage-based accounts of language change which allow individuals to modify their behaviour across their lifespan, not just in the childhood language acquisition period. With more opportunities for individual behaviour to change per unit time, these theories allow changes to propagate through large speech communities more quickly. If the bias towards the innovation (the selection strength, *s*) is close to zero and the innovation rate per interaction is also small, changes at the population scale can then occur at roughly the same rate in different speech communities.

In addition to small selection and innovation rates, this mechanism further requires a short memory lifetime in comparison to the lifetime of an individual (days or less, depending on social network structure). Taken at face value, such memory lifetimes may be considered unreasonably short. Here, we advise caution. First, a short memory does not imply that individual speakers are continually changing their behaviour: individual speakers can remain constant in their behaviour for as long as those around them do. If innovations rarely propagate, then most speakers will be exposed to existing conventions and continue to adhere to them, even though during a period of change they may alter their behaviour relatively quickly, albeit in small increments. There is some evidence that such changes can occur in older speakers as well as younger speakers, for example, in a study of Montreal French [12]. Meanwhile, research on priming [74, 75] shows that individual linguistic utterances can affect a speaker’s behaviour in interactions in the very short term before fading away. It would be worth understanding whether such effects could effect more permanent changes, for example, when a change is in progress in a speech community, as this might then imply a shorter *effective* memory time at the individual level than intuition grounded in everyday experience suggests.

The second mechanism that can reduce the sensitivity of grammatical change to population size are social network effects. Specifically, heterogeneous networks, in which a small number of well-connected speakers interact with a large number of poorly-connected speakers, lead to an effective population size (and therewith a characteristic timescale for change) that increases sublinearly with population size. Since this heterogeneity is a feature of certain social networks (e.g., those relating to phone calls, movie collaborations and social media [48–50]), it is reasonable to assume that this is a property of human social interactions more generally. It is interesting to note that sublinear relationships between rates of change and population sizes have been reported in other empirical studies of language change [64, 67]. Heterogeneous social networks offer one possible explanation for this phenomenon. To investigate this possibility further, it would be interesting to obtain more concrete information about the structure of linguistic interactions as well as how these stratify by age. If it were found, for example, that children’s networks are more homogeneous than adult’s, then this would point towards adults playing a key role in propagating an innovation throughout the speech community.

Although our statements about the relationship between individual behaviour and population-level change are grounded in a specific model of individual behaviour, we do not expect them to change if a different model was used. The reason for this is that any model that involves individual agents basing some or all of their future behaviour on that displayed by others (whether through learning or use) is expected to fall into the Wright-Fisher class [37]. The precise relationship between parameter values in the individual-based model and those in the population-level origin-fixation model may vary between models: however, in any two models with similar memory lifetimes, innovation biases and social network structures would be expected to have the same behaviour at the population scale. In S1 Appendix, we demonstrate this in the case of an extended model in which all properties vary between speakers, in which there is turnover in the population and social networks change over time.

This is not intended to imply that every feasible influence on language change is contained within the Wright-Fisher model used here (at least, at some level of abstraction). For example, we have excluded the possibility of a conformity bias [76, 77], wherein speakers suppress minority variants in favour of those in the majority. Such a bias however makes it increasingly difficult for innovations to propagate as the population increases in size, and therefore would be expected to exacerbate the problems of sensitivity to population size. We have also assumed that factors influencing individual linguistic behaviour are constant over space and time. Specifically, social factors like prestige effects have been excluded, and it would be interesting in future work to establish whether these lead more readily to plausible accounts of historical language change.

## Supporting information

S1 Appendix Details of data and methods. (PDF)

S1 Data Excel file containing historical population size estimates. (XLSX)
