# Supplementary material for: How individuals change language
Source: PLoS One. 2021 Jun 2;16(6):e0252582. doi: 10.1371/journal.pone.0252582 (PMC8172061; doi:10.1371/journal.pone.0252582)
Supplement: S1 Appendix — (PDF) [file pone.0252582.s001.pdf]

How individuals change language

R. A. Blythe and W. Croft

S1 Appendix. Details of data and methods

This Appendix sets out in detail the empirical data set on article grammaticalisation cycles that was used in the main text, and in particular how a population size for each language was estimated. We also provide the explicit mathematical expressions for the likelihood functions that were used in the analysis, and explain in detail how we match up the dynamics of the Wright-Fisher model (that applies at the individual level) to the origin-fixation model (that applies at the population level).

S1.1 Empirical data set

Our empirical data set of 52 languages is derived from 84 sources that document instances of article usage in different languages at different times. In Table S1.1 we summarise the stages of the grammaticalisation cycles for the definite and indefinite articles that have been observed, along with a historical time period that covers these observations. The quantities that enter the likelihood analysis are the total length of the historical period, and the number of changes in the article that occurred within it. In most cases, the beginning and end of each period corresponds to the earliest and most recent record (in cases where the language is still spoken, the latter is the present day). The exception to this is Hebrew, which was not spoken for a 1700-year period. In this case we take processes of change to be halted during this period. In cases where a language split into several daughter languages, we take the observation period for the parent language to end at the time of split, and the daughters' observation periods to begin. We also quote a measure of relative population size (the *weight* of a language), which can be converted into an estimate of the true population size using the procedure described in Section S1.2 below. These weights are obtained from geographical population sizes as described in Section S1.3 below. Finally in this table we record the relevant sources of historical language use so our characterisation of the historical data can be verified as required.

Language	Period	Definite	Indefinite	Weight	References
English	700CE – 2000CE	1,2	0,1,2	90.9	[1, 2]
German	200CE – 2000CE	0,1,2	0,1	286	[3, 4]
Common Scandinavian	1100CE – 1300CE	3	0	61.3	[5]
Icelandic	1300CE – 2000CE	3	0	1.00	[5]
Swedish	1300CE – 2000CE	3	0,1	29.3	[5, 6]
Irish	800CE – 1600CE	2	0	26.2	[7, 8]
Welsh	1100CE – 2000CE	2	0	1.85	[9, 10]
Greek	850BCE – 2000CE	1,2	0,1	55.0	[11–14]
Latin	100BCE – 500CE	0,1	0	948	[15–17]
French	500CE – 2000CE	1,2	0,1	366	[15, 16, 18]
Romanian	500CE – 2000CE	1,2,3	0,1	53.5	[18]
Bulgarian	800CE – 2000CE	0,1,2,3	0	32.8	[19, 20]
Russian	800CE – 2000CE	0	0	323	[21]
Egyptian	5000BCE – 700CE	2,3	0,1	181	[22–24]
Arabic	700CE – 2000CE	3	3,0	458	[25]

Language	Period	Definite	Indefinite	Weight	References
Hebrew	1200BCE – 200CE				
	1900CE – 2000CE	3	0	20.5	[26–28]
Persian	500BCE – 2000CE	0	0,1,2,3	130	[29–31]
Indo Aryan	700CE – 1000CE	0	0	2.51×10^3	[32]
Bengali	1000CE – 2000CE	0,1,2,3	0,1,2,3	590	[32]
Assamese	1000CE – 2000CE	0,1,2,3	0,1,2,3	88.5	[32]
Hindi	1000CE – 2000CE	0	0	737	[32]
Gujarati	1000CE – 2000CE	0	0	118	[32]
Korean	900CE – 2000CE	0	0	82.1	[33]
Japanese	700CE – 2000CE	0	0	231	[34]
Chinese	1000BCE – 2000CE	0	0	3.39×10^3	[35]
Classical Nahuatl	1500CE – 1600CE	2	1	22.1	[36, 37]
Tetelcingo Nahuatl	1600CE – 2000CE	2,3,0	1	5.53×10^{-3}	[38]
North Pueblo Nahuatl	1600CE – 2000CE	2	1,2	0.111	[39]
Michoacan Nahuatl	1600CE – 2000CE	2,3,0,1	1	3.32×10^{-3}	[40]
Huasteca Nahuatl	1600CE – 2000CE	2,3,0,1	1	1.31	[41]
Yucatec Maya	1450CE – 2000CE	1	0,1	0.918	[42, 43]
Quiche Maya	1400CE – 2000CE	1	1	2.15	[44, 45]
Cakchiquel Maya	1500CE – 2000CE	1,2	1	1.15	[46, 47]
Georgian	300CE – 2000CE	1,2,3,0	0	4.81	[48–50]
Armenian	400CE – 2000CE	2,3	0,1,2	7.21	[51, 52]
Aramaic	950BCE – 700CE	3,0	0	28.9	[53]
Geez	400CE – 1000CE	0	0	12.1	[54, 55]
Tigrinya	1000CE – 2000CE	0,1	0	10.6	[56, 57]
Tigre	1000CE – 2000CE	0,1,2	0,1	1.51	[58]
Akkadian	2000BCE – 600BCE	0	0	43.7	[59, 60]
Sumerian	3200BCE – 2000BCE	0	0	43.7	[61, 62]
Tamil	250BCE – 2000CE	0	0,1	184	[63–66]
Tibetan	800CE – 2000CE	0	0	13.5	[67–69]
Mongolian	1250CE – 2000CE	0	0	15.4	[70, 71]
Turkish	1200CE – 2000CE	0	1	173	[72, 73]
Khmer	800CE – 2000CE	0	0	32.4	[74–76]
Colonial Quechua	1600CE – 1700CE	0	0	42.7	[77]
Ayacucho Quechua	1700CE – 2000CE	0	0	2.59	[77, 78]
Imbabura Quechua	1700CE – 2000CE	0	0	0.683	[77, 79]
Huallaga Quechua	1700CE – 2000CE	0	0	0.0399	[77, 80]
Aymara	1600CE – 2000CE	0	0	8.55	[77, 81, 82]
Mapuche	1600CE – 2000CE	0	1	17.8	[77, 83, 84]

Table S1.1: Empirical dataset of historical language changes. Period: the historical periods over which observations were made. Definite, Indefinite: Stages of the grammaticalisation cycles observed for each article. Weight: relative historical average population size.

S1.2 Historical populations of geographical regions

We use a survey of population sizes across many regions of the world [85] to fit the following model for the size $N_i(t)$ of region i at time t, measured in years since 1BCE:

$$N_i(t) = w_i N_0 g(t) \ . \tag{S1.1}$$

In this model, N_0 sets an overall scale, w_i is a region-dependent *weight* that specifies its relative size, and $g(t)$ is a universal time-dependent growth function. This model amounts to an assumption that populations in different regions of the world maintain constant ratios over the relevant historical time period. Below we show that this model provides an estimate of a region's population size that is accurate to within a factor of 2.8 at a confidence level of 95%. The source data for this analysis is provided in the S2 File as an MS Excel spreadsheet; the resulting weights are specified in Table S1.2. Although not a particularly precise estimate, this variation of a factor of 2.8 is to be compared with a factor of 3400 variation in the weights themselves, and an overall increase by a factor of 300 in population sizes from 5000BCE to the present day. Consequently this simple model captures the range of population sizes and their changes over time rather well with a single parameter per geographical region, and we do not feel there would be much to be gained from using a more refined model.

Region	Weight	Region	Weight
Ancient Egypt	181	Ireland	26.2
Arabia	105	Italy	288
Austria	41.2	Japan	231
Bolivia	18.3	Khmer Republic	32.4
Bulgaria	32.8	Korea	82.1
C Turkestan Tibet	53.9	Libya	12.1
Caucasia	40.1	Maghreb	125
Chile	17.8	Mexico	111
China	3.39×10^3	Mongolia	11.4
Czechoslovakia	77.6	Nepal	56.0
Denmark	18.5	Norway	13.5
Ecuador	13.7	Pakistan India Bangladesh	2.95×10^3
Egypt	114	Palestine Jordan	20.5
England Wales	92.7	Peru	39.9
Ethiopia	50.3	Poland	84.7
France	366	Romania	53.5
Germany	245	Russia In Europe	323
Greece	55.0	Sri Lanka	32.0
Guatemala	14.3	Sweden	29.3
Iberia	241	Syria Lebanon	38.5
Iceland	1.00	Turkey In Asia	230
Iran	130	Yugoslavia	81.5
Iraq	43.7		

Table S1.2: Relative historical average size of each geographical region relevant to the languages in the sample (all to 3 s.f.). These sizes are normalized such that the smallest such region (Iceland) has a relative size of 1.

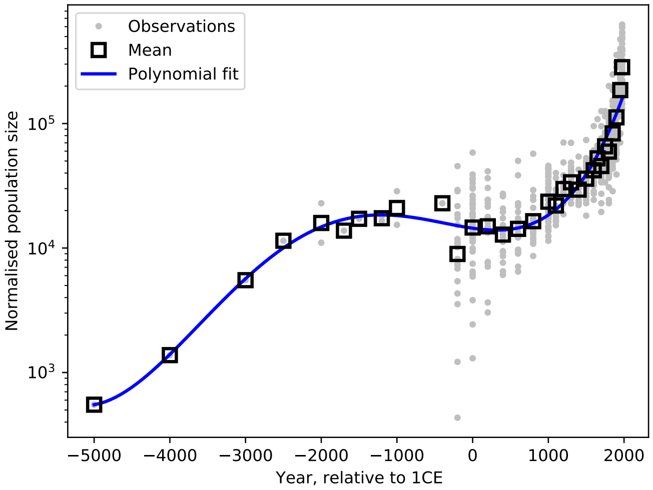

Figure S1.1: Population sizes normalised by by the weight factor w_i for each geographical region i. The grey dots show observations recorded in [85]. The black squares are the mean population size after normalisation has been applied to minimise the variance between different regions. The smooth line is a degree 4 polynomial fit to the logarithm of the mean normalised population size. See Table S1.3 for the coefficients in this polynomial.

The weights and the unknown function $g(t)$ are found by performing a linear least-squares fit to

$$\ln(N_i(t_j)) = \ln(w_i) + \ln(N_0) + \ln(g(t_j)) + \epsilon_i(t_j) \ , \tag{S1.2}$$

where $N_i(t_j)$ is the population size in region i at time point t_j as recorded in [85], and the parameters $a_i = \ln(w_i)$ and $b_j = \ln(g(t_j))$ are varied to minimise the sum of square residuals $\epsilon_i(t_j)$. This minimisation problem is underdetermined, and a unique solution is obtained after fixing $g(0) = 1$ and $w_i = 1$ for the smallest region in the sample (Iceland). This procedure yields an overall scale $N_0 = 14600$ (to 3 s.f.), and the weights w_i are presented in Table S1.2. In Figure S1.1, we plot the normalised population sizes $N_i(t_j)/w_i$, along with a fit to its mean, $g(t)$, whose logarithm is found to be well described by a quartic polynomial. This figure demonstrates that there is some scatter around this average, which we quantify further with the distribution of residuals that is shown in Figure S1.2. We find that although this distribution is not normal, the central 95% of the residuals span the interval from -1.02 to 1.3. Since these residuals are natural logarithms, this range corresponds to the overall factor of 2.8 error (in either direction) on the estimated population size, as claimed above. The R^2 statistic for the linear least-squares fit is 0.923 (to 3 s.f.).

Coefficient	c_0	c_1	c_2	c_3	c_4
Value	-0.0127	-2.00×10^{-4}	2.13×10^{-7}	2.04×10^{-10}	2.55×10^{-14}

Table S1.3: Coefficients in the polynomial fit $c_0 + c_1 t + c_2 t^2 + c_3 t^3 + c_4 t^4$ to the function $\ln(g(t))$ in (S1.2) obtained by least-squares minimisation.

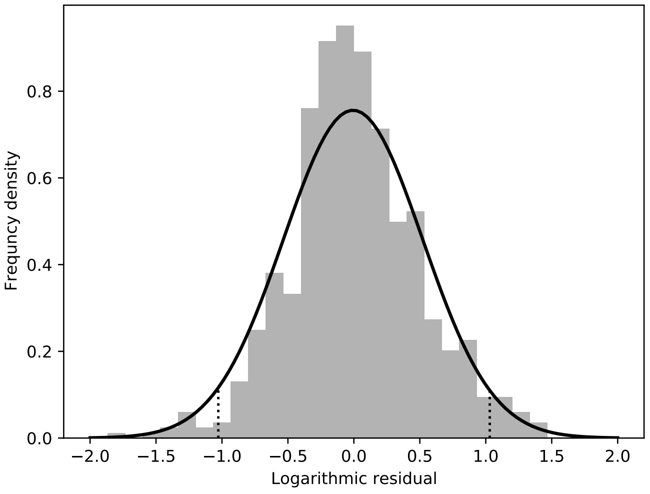

Figure S1.2: Distribution of the logarithmic residuals, $\ln(N_{\mathrm{obs}}/N_{\mathrm{fit}})$ where N_{obs} and N_{fit} are the observed and fit population sizes, respectively. Solid line: normal distribution with the same mean and variance. Dashed lines: extent of the central 95% of the residuals.

S1.3 Geographical composition of languages

We assume that language sizes are linear combinations of the sizes of the geographical regions where they are spoken, and hence that these fractions remain constant over time. These combinations are provided in Table S1.4, with the resulting language sizes in Table S1.1. For most cases, we assume a one-to-one relationship between a geographical region (e.g., Sweden) and a language (Swedish). In other cases, further explanation is necessary:

- *English and Welsh* — Currently, around 5% of the population of England and Wales resides in Wales. Assuming this fraction to be constant over history, and that roughly one third of Welsh residents are speakers of Welsh, we arrive at a 98% to 2% split across England and Wales into English and Welsh speakers respectively.
- *Scandinavian* — Two of the languages in the sample, Icelandic and Swedish, descended from Common Scandinavian with the split estimated to have occurred around 1300CE. We therefore take the period from 1100CE to 1300CE as common to both languages, and to have a correspondingly larger population (see below for details of how this was incorporated into the analysis).
- *Irish* — Until around 1600CE, we assume the entire population of Ireland is Irish-speaking before the Irish-speaking population declines. We therefore truncate the time-window for Irish in the analysis at 1600CE, rather than the present day (see Table S1.1).
- *Latin* — Analogously to the Scandinavian languages, we take Latin to be an ancestor of French and Romanian, estimating the split to take place around 500CE, before which both daughter languages are considered to have a common history.
- *Turkish* — The region designated Turkey-in-Asia by McEvedy and Jones [85] includes a significant population of Kurdish, Armenian and Greek speakers. We take the number of Turkish speakers to be 75% of this larger population.
- *Hebrew* — Hebrew was not spoken between around 200CE and 1900CE, being a liturgical and written language in the intervening period. Here, we assume that the language was frozen (unable to change) in the period that it was not spoken.
- *Aramaic* — McEvedy and Jones [85] combine Syria (where Aramaic was spoken) and Lebanon into a single region. We estimate that Aramaic speakers make up 75% of this region.
- *Ethiopian Semitic* — Ge‘ez is assumed to be the direct ancestor of both Tigré and Tigrinya, with a split occouring at 1000CE. The fraction of Ethiopia in which each daughter language is spoken is assumed to be constant and equal to 1986 values taken from [86].
- *Indo-Aryan* — Masica [32, p8] quotes a figure of 640m total speakers of Indo-Aryan languages. The majority of these reside in the region designated as Pakistan, India and Bangladesh by McEvedy and Jones [85], for which the most recent estimate of population size (1975CE) was given as 745m. We therefore assume the population of Indo-Aryan speakers to track the size of Pakistan, India and Bangladesh, but scaled by a factor of 85% to match recent estimates in [86]. We take the Indo-Aryan ancestor language to split at around 1000CE; the daughter languages Bengali, Assamese, Hindi and Gujarati that were included in the sample are thereby taken to share a common period of evolution from around 700CE to 1000CE (as with the Scandinavian and Latin languages). These sizes of each of these daughter languages

were also taken to track the population of Pakistan, India and Bangladesh, again using the ratio that applies to the present-day populations.

- *Tamil* — Tamil is spoken in India and Sri Lanka. Proportions of the corresponding regions documented by McEvedy and Jones [85] were estimated using numbers for 1986CE in [86].
- *Tibetan* — We take 25% of the population of the Chinese Turkestan and Tibet region [85] to be Tibetan.
- *Mongolian* — The Mongolian-speaking population extends beyond the region designated as Mongolia by McEvedy and Jones which excludes Inner Mongolia. We assume that the total population of Mongolian speakers is 35% larger than that of Mongolia.
- *Mesoamerican languages* — These languages are spoken in regions designated as Mexico and Guatemala by McEvedy and Jones [85]. We take current estimates of their speaker numbers as fractions of the relevant geographical regions to obtain the weight of the languages, and a historical estimate to determine the fraction of Mexico population that spoke Classical Nahuatl before splitting into daughters at around 1600CE. However, given the recent decline (in particular) of Nahuatl as it was replaced by Spanish in Mexico, this means that the numbers post-split are likely to be underestimates, and furthermore it may not be reasonable to assume that the speaker numbers are a constant fraction of the geographical population over time. However, we do not believe that this uncertainty greatly affects our results.
- *Quechua* — We consider three varieties of Quechua, all of which are taken to be descendants of a common Colonial Quechua language spoken widely across Bolivia, Ecuador and Peru before 1700CE. After this split, we assume that the speakers of the daughter varieties are constant fractions of Peru and Ecuador set at values that pertain to the 1970s [86].

Language	Geographical composition
Akkadian	100% Iraq
Arabic	100% Arabia + 100% Iraq + 100% Palestine Jordan + 100% Syria Lebanon + 100% Maghreb + 100% Libya + 100% Egypt
Aramaic	75% Syria Lebanon
Armenian	18% Caucasia
Assamese	3% Pakistan India Bangladesh
Ayacucho Quechua	6.5% Peru
Aymara	25% Bolivia + 10% Peru
Bengali	20% Pakistan India Bangladesh
Bulgarian	100% Bulgaria
Cakchiquel Maya	8% Guatemala
Chinese	100% China
Classical Nahuatl	20% Mexico
Colonial Quechua	50% Bolivia + 100% Ecuador + 50% Peru
Common Scandinavian	100% Denmark + 100% Sweden + 100% Norway
Egyptian	100% Ancient Egypt
English	98% England Wales
French	100% France
Geez	24% Ethiopia
Georgian	12% Caucasia

Language	Geographical composition
German	100% Germany + 100% Austria
Greek	100% Greece
Gujarati	4% Pakistan India Bangladesh
Hebrew	100% Palestine Jordan
Hindi	25% Pakistan India Bangladesh
Huallaga Quechua	0.1% Peru
Huasteca Nahuatl	1.18% Mexico
Icelandic	100% Iceland
Imbabura Quechua	5% Ecuador
Indo Aryan	85% Pakistan India Bangladesh
Irish	100% Ireland
Japanese	100% Japan
Khmer	100% Khmer Republic
Korean	100% Korea
Latin	100% France + 100% Iberia + 100% Italy + 100% Romania
Mapuche	100% Chile
Michoacan Nahuatl	0.003% Mexico
Mongolian	135% Mongolia
North Pueblo Nahuatl	0.1% Mexico
Persian	100% Iran
Quiche Maya	15% Guatemala
Romanian	100% Romania
Russian	100% Russia In Europe
Sumerian	100% Iraq
Swedish	100% Sweden
Tamil	6% Pakistan India Bangladesh + 23% Sri Lanka
Tetelcingo Nahuatl	0.005% Mexico
Tibetan	25% C Turkestan Tibet
Tigre	3% Ethiopia
Tigrinya	21% Ethiopia
Turkish	75% Turkey In Asia
Welsh	2% England Wales
Yucatec Maya	0.83% Mexico

Table S1.4: Geographical composition of the speech community for each language in the sample. The resulting relative historical average population sizes are given in Table S1.1.

In the main text, the model calls for a single size to characterise each population over the relevant historical period. We use the mean of $N_i(t)$ given by Eq. (S1.1) over the historical time period (or periods, for Hebrew) given in Table S1.1, summed over regions i with the weights given in Table S1.4.

S1.4 Origin-fixation model

As described in the main text, we have constructed an origin-fixation model to describe the dynamics of language change at the population level, and more specifically provide likelihood functions

for the empirical data sets under various assumptions on the underlying linguistic behaviour of individuals. In this model, an innovation (mutation) of type $i+1$ that successfully propagates through (invades) a population of type i individuals is introduced as a Poisson process with rate ω_i. Recall from the main text that we refer to the introduction of a successful innovation to the population as an *origination* event. We take $\omega_i = \bar{\omega}/(V f_i)$, where f_i is the typological frequency of variant $i = 1, 2, \ldots, V$ across the world's languages, so that mean time between the onset of origination events is proportional to f_i. In most origin-fixation models [87], the fixation time T_F is idealised as zero. As explained in the main text, in language change the timescale of fixation (decades to hundreds of years) is not greatly separated from the origination timescale (hundreds to thousands of years). Consequently, we must generalise to nonzero fixation times. Specifically, we model the fixation process as one whose time to fixation is drawn from a Gamma distribution (this because the fixation time is necessarily positive, and we wish to treat its mean and variance as independent quantities). Since in this framework it is possible that the change to the next stage of the cycle may be triggered before the previous one has gone to fixation, we must also account for interference between successive originations.

S1.4.1 Likelihood function

In the main text, we compared different models using the Akaike Information Criterion, defined through equation (4). This involves the likelihood function

$$\mathcal{L} = \prod_{i=1}^{n} \mathcal{L}_{m_i}(t_i) \tag{S1.3}$$

where $\mathcal{L}_{m_i}(t_i)$ gives the probability that exactly m_i language changes have occurred in a time window of length t_i these corresponding to language i in the sample. In this section, we explain how $\mathcal{L}_{m_i}(t_i)$, and therewith $\mathcal{L}$, is calculated.

We assume that at the beginning of an observation window, $t = 0$, only one variant is present in the population. Let ω_1 be the origination rate at the first stage in the cycle and $\overline{T_F}$ and $\sigma^2_{T_F}$ be the mean and variance of the time for the innovation to reach fixation in the population, conditioned on this event occurring. Even in those cases where exact results are available [88], the functional form of the distribution $p_1(t)$ that the invading mutant fixes at time t is very complicated. We have found it is well approximated by the convolution of a Poisson process with rate ω and a Gamma distribution with mean $\overline{T_F}$ and variance $\sigma^2_{T_F}$ (see S1.5.2.1 below). That is

$$p_1(t) \approx \omega_1 \mathrm{e}^{-\omega_1 t} * \frac{(\beta t)^{\alpha-1} \beta \mathrm{e}^{-\beta t}}{\Gamma(\alpha)} \tag{S1.4}$$

where $\Gamma(\alpha)$ is the Gamma function and $*$ denotes the convolution operation. The parameters α and β are related to the mean and variance of the fixation time via

$$\overline{T_F} = \frac{\alpha}{\beta} \quad \text{and} \quad \sigma^2_{T_F} = \frac{\alpha}{\beta^2} \, . \tag{S1.5}$$

Once the innovation has gone to fixation, the next innovation can then be introduced to the population at rate ω_2 (which need not equal ω_1) and its fixation time is assumed to have the same mean and variance as the first mutant. The probability $P_m(t)$ that *at least* m changes have occurred by time t is obtained by convolving $p_1(t)$ with itself m times, and integrating from 0

to t. The probability $\mathcal{L}_m(t)$ that *exactly* m changes have occurred by time t is then given by $\int_0^t [P_m(t') - P_{m+1}(t')]\,\mathrm{d}t'$. This is most conveniently written in the form of the Laplace transform

$$\hat{\mathcal{L}}_m(s) = \frac{1}{s}\left(\prod_{i=1}^{m} \frac{w_i}{w_i+s}\right)\left(\frac{\beta}{\beta+s}\right)^{m\alpha}\left[1 - \frac{w_{m+1}}{w_{m+1}+s}\left(\frac{\beta}{\beta+s}\right)^{\alpha}\right] . \tag{S1.6}$$

Note that in the case $m = 0$, the product over i is set equal to unity.

It is possible to invert the Laplace transform and obtain explicit expressions for $\mathcal{L}_m(t)$ involving alternating sums of incomplete Gamma functions. Unfortunately, these expressions are difficult to compute to the desired numerical precision, due to cancellations between terms at the leading order. A much better approach is to numerically invert (S1.6) using the Euler algorithm as set out in [89]. This involves computing the sum

$$\mathcal{L}(t) \approx \frac{1}{t}\sum_{i=1}^{n} c_i \mathrm{Re}\hat{\mathcal{L}}_m\left(\frac{\zeta_i}{t}\right) \tag{S1.7}$$

where the number of terms n, the weights c_i and the nodes ζ_i depend on the desired precision [89]. We have found five digits of precision sufficient for our needs, which corresponds to $n = 18$, a remarkably small number of function evaluations given the complexity of the problem. The different cultural evolutionary scenarios that we test in the main text give rise to a wide range of different parameter combinations. To maintain numerical precision in calculating the logarithm of the likelihood $\mathcal{L}_m(t)$ across the full range of parameter values, a few minor modifications to the standard Euler algorithm were required:

- For the case $m = 0$, $\mathcal{L}_0(t) \to 1$ as $t \to 0$. Here, the log likelihood is close to zero, and therefore for this to be obtained to the desired precision, we invert the transform of $1 - \mathcal{L}_0(t)$ (which is close to zero), and use a library function to evaluate $\ln(1-x)$ for small x.
- In all other cases, the likelihood has a leading exponential decay

  $$\mathrm{e}^{-s^* t} \quad \text{where} \quad s^* = \min\{\beta, \omega_1, \ldots, \omega_m\}$$

  is the location of the singularity in (S1.6) closest to the origin in the complex-s plane. Since the combination s^*t can become large (leading to a very small likelihood), we maintain precision via the identity $\ln \mathcal{L}(t) = -s^*t + \ln \mathcal{R}_{s^*}(t)$ where $\mathcal{R}_{s^*}(t)$ is the inverse of the shifted Laplace transform $\hat{\mathcal{L}}(s - s^*)$. Note that the apparent pole at $s = 0$ is cancelled by a zero in the numerator, which permits this shift of the integration contour.
- Finally, some of the terms in the sum (S1.7) can take values that are sufficiently small or large to cause overflow when working to machine precision. We handle this by computing the logarithm of each term in the sum and subtracting out the largest real part from all terms. Then the remainders can be safely exponentiated and summed without causing overflow. The contribution that was subtracted is then reinstated into the result for the log likelihood at the end of the calculation.

The likelihood of the macroscopic Poisson process with state-dependent origination rates $\omega_1, \omega_2, \ldots$ can be obtained from the inversion of (S1.6) after taking the limit $\beta \to \infty$. A complete implementation of the likelihood analysis code is provided for reference at [90].

S1.4.2 Interference correction

The calculation of the likelihood function above is conditioned on each origination going to fixation before the next origination event is triggered. When the origination rate ω_i is comparable to $1/\overline{T_F}$, successive origination events can interfere, which ultimately leads to coexistence of multiple variants rather than a sequence of changes going through the population. This feature is inconsistent with the empirical data, in which the typical situation is that one convention dominates, or that we are in transition from one stage of the cycle to the next. To prevent a maximum of the likelihood function being found in this region, we need to multiply it by the probability that an originated innovation *does* go to fixation: this then gives us the joint probability that fixation will occur, and that it has occurred by a given time.

We introduce therefore the correction C_i, which is the propbability that the innovation introduced at stage i (i.e., the one that precipitates a change to stage $i+1$) goes to fixation without interference. Then,

$$\mathcal{L}'_m(t) = \left(\prod_{i=1}^{m} C_i\right) \mathcal{L}_m(t) \; . \tag{S1.8}$$

In the case of the Poisson process (or the Wright-Fisher model with infinite selection) $T_F \equiv 0$, and so no correction is needed ($C_i \equiv 1$). In the general Wright-Fisher model, we find from numerical computations that C_i is well approximated by $C_i = \mathrm{e}^{-\omega_i \overline{T_F}}$ (see S1.5.2.1 below). However, the precise form of the correction is not too important in terms of likelihood maximisation, as long as $C_i \approx 1$ when innovations can propagate freely without interference, and decreases towards 0 when they cannot.

S1.5 Wright-Fisher model

At the individual speaker level, we use a Wright-Fisher model. We provide the full definition of this model here, explain how we extract from it the parameters α, β and ω_i in the origin-fixation model, and demonstrate numerically that the latter serves as a good approximation to the Wright-Fisher model after averaging over individual innovation frequencies.

S1.5.1 Definition

The dynamics of the Wright-Fisher model are illustrated in Fig. 1 of the main text. We assume that at a given point in time, the language is in transition from stage i to $i+1$ of the cycle. Each of the N speakers is then characterised by the frequency x_n that they use the innovation (i.e., produce an utterance consistent with stage $i+1$ of the cycle). Each speaker updates their grammar (their x_n value) at intervals of $\Delta t = 1/R$, where R is the interaction rate. They retain a fraction $(1-\epsilon)$ of their existing grammar, and replace the remaining fraction with a memory of either the innovation or the existing convention, this depending on the relevant linguistic interactions they have over the interval Δt. If we define a variable τ_n such that $\tau_n = 0$ when the speaker stores a memory of the convention, and $\tau_n = 1$ for the case of the innovation, we have

$$x'_n = (1-\epsilon)x_n + \epsilon\tau_n \; . \tag{S1.9}$$

The probability p_n that $\tau_n = 1$ depends on the frequency of the innovation in the speaker's neighbourhood, $\bar{x}_n$, the individual innovation rate η and the selection strength s. The specific prescription

is

$$p_n = \frac{1-\bar{x}_n}{1+\bar{x}_n s}\eta_i + \frac{(1+s)\bar{x}_n}{1+\bar{x}_n s} \, . \tag{S1.10}$$

In words, this equation says that a speaker first samples an instance of linguistic behaviour from their local neighbourhood, wherein the convention is given weight 1, and the innovation weight $1+s$. That is, if $s > 0$ the innovation is selected for; and if $s < 0$ it is selected against. If this instance of linguistic behaviour corresponds to the convention, there is a probability η_i that it is recorded by the speaker as the innovation (i.e., a mutation from stage i to $i+1$ of the cycle has occurred at the individual level).

We are deliberately abstract in our specification of the model, as different mechanisms can give rise to selection and innovation. A number of concrete examples, and their relation to linguistic theories, are provided in the main text.

S1.5.2 Correspondence with the origin-fixation model

To connect the Wright-Fisher model to the origin-fixation model, we need to work out the values of the parameters ω_i, $\overline{T_F}$ and $\sigma^2_{T_F} \equiv \overline{T_F^2} - \overline{T_F}^2$ of the latter that are implied by the former. We consider first of all the origination rates. Starting from a state with $x_n = 0$ for all speakers, we see that each agent has a probability η_i of generating an innovation at a rate R. Then the total rate at which successful innovations propagate is $NR\eta_i Q(\epsilon/N)$, where $Q(x_0)$ is the probability that an innovation with frequency x_0 in the *population* goes to fixation. $x_0 = \epsilon/N$ because we assume the innovation rate is sufficiently small that exactly one speaker starts off with the innovation at level ϵ. Within this low innovation-rate regime, the innovation then propagates under the influence of selection and drift (fluctuations arising from the finite exposure to linguistic behaviour) until it reaches fixation.

To calculate $Q(x_0)$, we first define $\delta x_n = x'_n - x_n$ and determine the expectation values $\langle \delta x_n \rangle$ and $\langle (\delta x_n)^2 \rangle$ over the distribution of τ_n given above. This allows one to write down the forward or backward Kolmogorov equation for the set of individual speaker frequencies x_n via the Kramers-Moyal expansion. Various studies, e.g., [91–93], have shown that for an appropriate weighted average x of these individual speaker frequencies, one has the backward Kolmogorov equation

$$T_M \frac{\partial q(t|x)}{\partial t} = sx(1-x)\frac{\partial q(t|x)}{\partial x} + \frac{1}{2N_e}x(1-x)\frac{\partial^2 q(t|x)}{\partial x^2} \tag{S1.11}$$

for the probability distribution $q(t|x)$ that an innovation with initial frequency x reaches fixation at time t. In this equation, s is as defined in the Wright-Fisher model, $T_M = 1/(R\epsilon)$ is the memory lifetime identified in the main text, and N_e is an effective population size

$$N_e = \frac{N}{\epsilon}\frac{\overline{z}^2}{\overline{z^2}} \, . \tag{S1.12}$$

The quantity z_n is defined as the number of others speakers that speaker n can observe the linguistic behaviour of; we assume that each of these speakers is given the same amount of attention, although the number of neighbours z_n can vary across a social network. (Derivations of this result can be found in [91–93]).

This Kolmogorov equation is extremely well studied in the population genetics literature (e.g. [88, 94]). In particular, the procedure for obtaining moments of the time to reach fixation from a single

mutant ($x \to 0$), conditioned on fixation occurring, is well established [94]. To compute $\overline{T_F}$ and $\sigma^2_{T_F}$, we require the first two moments. Defining

$$F_k(x) = \int_0^\infty t^k q(t|x)\,\mathrm{d}t\ , \tag{S1.13}$$

we have

$$Q(x) = F_0(x)\ , \quad \overline{T_F} = \lim_{x\to 0} \frac{F_1(x)}{F_0(x)} \quad \text{and} \quad \overline{T_F^2} = \lim_{x\to 0} \frac{F_2(x)}{F_0(x)} \tag{S1.14}$$

where $Q(x)$ is the probability that a mutant with initial frequency x fixes. By multiplying (S1.11) by t^k and integrating [94], we find the recursion

$$2N_e s x(1-x)F_k'(x) + x(1-x)F_k''(x) = -2kN_e T_M F_{k-1}(x) \tag{S1.15}$$

where a prime denotes differentiation. The boundary conditions of this differential equation are $F_k(0) = 0$ for all k, $F_0(1) = 1$ and $F_k(1) = 0$ for $k > 0$. For the case $k = 0$, the solution has the closed form [94]

$$Q(x) = F_0(x) = \frac{1 - \mathrm{e}^{-2N_e s x}}{1 - \mathrm{e}^{-2N_e s}}\ . \tag{S1.16}$$

Substituting $x = \frac{\epsilon}{N}$ we arrive at Eq. (3) in the main text. Although no general closed-form expression exists for the case $k > 0$, (S1.15) can still be integrated to obtain [94]

$$F_k(x) = 2kN_e T_M \left[(1 - Q(x)) \int_0^x \frac{F_{k-1}(y)}{y(1-y)} \frac{Q(y)}{Q'(y)}\,\mathrm{d}y + Q(x) \int_p^1 \frac{F_{k-1}(y)}{y(1-y)} \frac{1-Q(y)}{Q'(y)}\,\mathrm{d}y\right] \tag{S1.17}$$

which can be evaluated numerically and substituted into (S1.14) to obtain the desired moments.

The complexity of this numerical problem is simplified slightly by noting that

$$\overline{T_F^k} = \lim_{x\to 0} \frac{F_k(x)}{F_0(x)} = 2kN_e T_M \int_0^1 \frac{F_{k-1}(y)}{y(1-y)} \frac{1-Q(y)}{Q'(y)}\,\mathrm{d}y = \frac{kT_M}{s} \int_0^1 \frac{F_{k-1}(y)}{y(1-y)} \left[1 - \mathrm{e}^{-2N_e s(1-y)}\right] \mathrm{d}y \tag{S1.18}$$

and the fact that $\overline{T_F^k}$ is symmetric in $s \to -s$. However, we find that a numerical integration routine, implemented naïvely, becomes unreliable for small and large $N_e|s|$. In these regimes it both more efficient and less susceptible to numerical instability to use Taylor series and asymptotic expansions, respectively. Specifically, for small $|s|$ we use the approximations

$$\overline{T_F} \approx 2N_e T_M \left[1 - \frac{(2N_e s)^2}{72}\right] \qquad \text{when } 2N_e|s| < 10^{-3} \tag{S1.19}$$

$$\overline{T_F^2} \approx 8N_e^2 T_M^2 \left[\frac{\pi^2}{3} - 2 + \left(\frac{\pi^2}{36} - \frac{17}{54}\right)(2N_e s)^2\right] \qquad \text{when } 2N_e|s| < 10^{-2} \tag{S1.20}$$

and for large $|s|$

$$\overline{T_F} \sim \frac{2T_M}{|s|} \left[\ln(2N_e|s|) + \gamma - \frac{1}{2N_e|s|}\right] \qquad \text{when } 2N_e|s| > 500 \tag{S1.21}$$

$$\overline{T_F^2} \sim \frac{2T_M^2}{s^2} \left[4(\ln(2N_e|s|) + \gamma)^2 + \frac{\pi^2}{3}\right] \qquad \text{when } 2N_e|s| > 500 \tag{S1.22}$$

in which γ is the Euler-Mascheroni constant $\gamma = 0.577\ldots$.

S1.5.2.1 Numerical test of the correspondence between the models

In arriving at the Kolmogorov equation (S1.11), we made a number of approximations, in particular, that innovation (mutation) can be ignored when estimating the time to fixation. We therefore compare numerical solutions for the probability $P(t)$ that an innovation has reached fixation by time t within the full Wright-Fisher dynamics against the formulæ we have derived for the corresponding origin-fixation model. These results are shown in Fig. S1.3, and we find the essential features are well captured. Of particular importance are deviations from Poisson behavior that are evident at early times. These deviations arise from the fact that an innovation takes a finite amount of time to propagate through the whole population, and are absent in classical origin-fixation models where T_F is assumed to be zero. We see that even in a population of 100 speakers, the probability that a single change has occurred is suppressed for a historically relevant time (~100 generations, which would equate to $2,500$ years in a child-based model). It is this property that is ultimately responsible for the very low likelihoods that are encountered in the main text.

These numerics also allow us to estimate the form of the interference correction C_i introduced in S1.4.2. To achieve this, we consider a generalisation to the Wright-Fisher model where two innovations can occur. If the innovation rate is fast enough, the second innovation can occur before the first has gone to fixation. A plot of the probability that the first goes to fixation for a variety of innovation rates gives us the correction factor C_i. We find that the numerical data are reasonably well fit by the function $C_i e^{-\omega_i \overline{T_F}}$. As noted previously, it is not important to capture the exact form of this function, as the regime where successive innovations interfere is not empirically relevant. What is important is establishing where the correction becomes significant.

S1.5.3 Robustness of the Wright-Fisher model

It is well established in the population genetics literature (e.g. [95]) that the Wright-Fisher model approximates well the behaviour of a large number of evolutionary processes. In the main text, we presented one specific member from this class, since this allowed us to ascribe concrete meanings to the parameters N_e, s and T_M in terms of individual linguistic behaviour. Here, we demonstrate that many aspects of that specific model—for example, that all individuals have the same memory lifetime and that this remains constant over time, that they are equally biased in favour (or against) an innovation or do not themselves undergo processes of birth and death—are incidental. The key property is that linguistic behaviour is socially learnt, i.e., acquired from other members of the speech community, perhaps in the presence of biases.

This demonstration model comprises the following components:

- *Agent lifetime* — After being introduced into the population, an agent has a lifetime d drawn from a Gamma distribution with mean μ_d and variance σ_d^2. It is then removed from the population after time d has elapsed. If this removal causes the entire population to go extinct, a new population comprising a single individual is immediately established. (The probability of this event is very small once a steady state is reached, and is included simply to guarantee that every run of the simulation reaches the steady state).

- *Agent reproduction* — When an agent is introduced into the population, it is also assigned a number of offspring $k \geq 0$ drawn from a Geometric distribution with mean μ_k. Associated with each offspring $n = 1, 2, \ldots, k$ is the age b_n of the parent when the offspring is born. This age is drawn from a Gamma distribution with mean μ_b and variance σ_b^2. (If this age

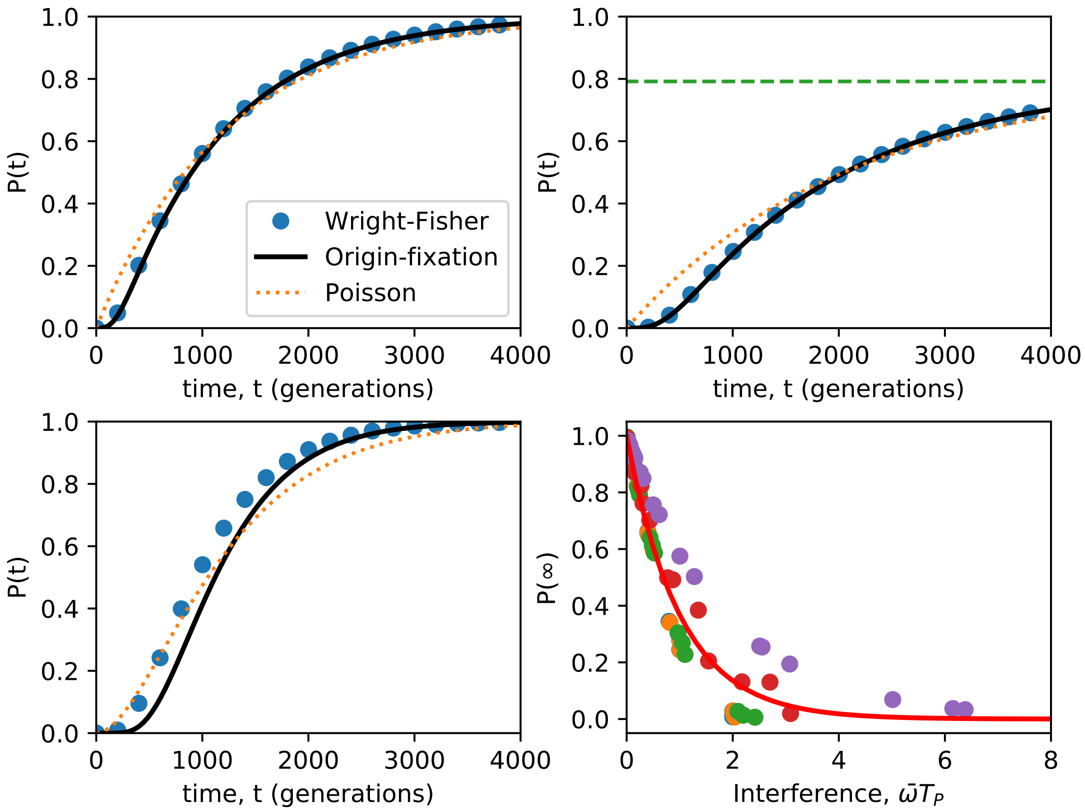

Figure S1.3: Comparison of the origin-fixation model with numerical solutions of the full Wright-Fisher model. (Top left) Probability that a single change has occurred, $N = 100$, $\eta = 10^{-3}$, $s = 0$. The dotted line is a Poisson process with the same mean. (Top right) Probability that the first of a sequence of two changes has occurred, $N = 200$, $\eta = 5 \times 10^{-4}$, $s = 10^{-3}$. Interference between the first and second innovation means that first change does not always go to fixation. (Bottom left) Probability that the second in a sequence of two changes have occurred, $N = 100$, $\eta = 10^{-3}$, $s = 10^{-2}$. (Bottom right) Probability that the first of two changes goes to fixation as a function of the interference $I = \omega\overline{T_F}$. This is reasonably well fit by the exponential $P(\infty) = \mathrm{e}^{-I}$.

exceeds d, the parent's age of death, then it is discarded and the distribution resampled). When the time of birth arrives, a new agent is entered into the population with probability 1 if the current population size N is smaller than a *carrying capacity* K, and with probability $1 - \frac{K}{N\mu_k}$ otherwise. This rule prevents an unbounded exponential growth of the population, and in practice causes its size N to fluctuate around K in the steady state.

- *Initial interaction network* — When an agent is born, its parent is marked as an *interlocutor* (that is, someone who may influence its linguistic behaviour). The offspring also inherits each of its parent's z interlocutors with probability $\frac{\mu_i}{z}$. The offspring inherits all interlocutors if $\mu_i > z$; otherwise it inherits μ_i of its parent's interlocutors on average.

- *Expansion of the interaction network* — At the time of birth, an agent is also assigned an age e drawn from a Gamma distributions with mean μ_e and variance σ_e^2. At this age, each member of the population, n, is assigned a weight $w_n = w_0$ if they are one of the agent's existing interlocutors, or $w_n = \exp(-h|\delta|)$ where δ is the age difference between the two agents. The existing interlocutors are then discarded, and a new set built up, with agent n being marked as an interlocutor with probability $\mu_i' w_n / Z$ (capped at 1) where $Z = \sum_n w_n$. Here μ_i' is the mean number of interlocutors arising from the expansion of the interaction network. If the *homophily* parameter $h = 0$, then every agent in the population has the same chance of becoming an interlocutor at the time of expansion; for $h > 0$, agents who are closer in age are more likely to interact after expansion than those further away in age. Thus, in this model, young agents tend to be influenced by their parents (and their parent's peer group) whereas older agents tend to influenced by their own peer group.

- *Initial linguistic behaviour* — When an agent is created, the frequency x with which it uses the innovation is inherited unchanged from its parent.

- *Rate of linguistic interactions* — An agent participates in a linguistic interaction that modifies its behaviour at age a according to a time-inhomogeneous Poisson process of intensity $R(a)\mathrm{d}a = R_\infty + (R_0 - R_\infty)\mathrm{e}^{-a/\theta}$. That is, when the agent is born, the rate of (behaviour-modifying) interactions is R_0, and this decays exponentially to $R_\infty < R_0$ with characteristic timescale θ. Thus, in this model, an agent may become less liable to change their behaviour as they age. Each of the parameters R_0, R_∞ and θ are assigned from distributions when the agent is born. R_0 is drawn from a Gamma distribution with mean μ_R and variance σ_R^2. $R_\infty = R_0/(1 + r)$ where r is drawn from a Gamma distribution with mean μ_r and variance σ_r^2. The decay time θ is drawn from a Gamma distribution with mean μ_θ and variance σ_θ^2.

- *Behaviour modification in a linguistic interaction* — When a linguistic interaction takes place, each of the agent's interlocutors is included in the interaction with probability q, subject to a constraint that at least one interlocutor must be present. The mean frequency of the innovation among this subset of interlocutors, y, is calculated. The agent then updates their innovation frequency x as described in the main text: a fraction ϵ of their existing frequency is replaced with $\tau = 1$ if they perceive the innovation in the interaction, and with $\tau = 0$ if they perceive the convention. The probability that $\tau = 1$ is $\frac{(1+\chi)y}{1+\chi y}$ where χ is a bias towards (or against) the innovation. Both the parameters ϵ and χ are randomly assigned to an agent at birth. ϵ is drawn from a Beta distribution on $[0, 1]$ with mean μ_ϵ and variance σ_ϵ^2. The bias χ is drawn from a normal distribution with mean μ_χ and variance σ_χ^2.

It is evident that this model is much more complex than that described in the main text: agents are born and die, and the population size fluctuates over time; there is vertical and horizontal

transmission of linguistic behaviour, the proportion of which changes during an agent's lifespan; agents can be more or less liable to changing their behaviour, and the rate at which they do so decreases over time; and they can be more less disposed to the innovation. It also has a correspondingly increased number of parameters: 23 in total; by contrast the Wright-Fisher model described in the main text has only 5 (if one excludes, as here, the possibility of an innovation being generated during the process of fixation).

To establish that the demonstration falls into the general class of Wright-Fisher models—and can be well represented with a smaller number of parameters—we use the fact that over short time increments Δt, we should find that the first two moments in the change in the frequency of the innovation over the population, Δx, are

$$\overline{\Delta x} = \frac{\Delta t}{T_M} s x(1-x) \quad \text{and} \quad \overline{\Delta x^2} = \frac{\Delta t}{T_M}\frac{1}{N_e} x(1-x) \, , \tag{S1.23}$$

where x is the innovation frequency, and the parameters T_M, s and N_e are as described in the main text. Here, the overlines denote averages over multiple time intervals. The crucial point is that correspondence with the Wright-Fisher model is manifested as both moments varying with frequency as $x(1-x)$.

In Figure S1.4 we plot these moments obtained from simulations as a function of x under three choices of the parameters controlling the bias χ: (i) $\mu_\chi = 0$, $\sigma_\chi = 0.005$; (ii) $\mu_\chi = 0.005$, $\sigma_\chi = 0$; and (iii) $\mu_\chi = 0.005$, $\sigma_\chi = 0.005$. That is, in case (i) agents are equally likely to be biased in favour of, or against the innovation; in case (ii) all agents are biased by the same amount in favour of the innovation; and in case (iii) agents are more likely to be biased in favour of, rather than against, the innovation. The values of the remaining 21 parameters are given in Table S1.5 (and were chosen to be in the range that could plausibly describe a small human-like population). Fits of the parabola $Ax(1-x)$, with the amplitude A as a free parameter, are shown as dashed lines if Figure S1.4. We find these empirical fits describe well the jump moments obtained from simulation (albeit subject to some noise in the estimation of the first jump moment, which is likely a consequence of the wide variation in individual behaviour this demonstration model permits).

Parameter	Meaning	Value
K	Carrying capacity	1000
μ_k	Mean number of offspring	2.0
μ_b	Mean age of parent at offspring birth	30.0yr
σ_b	Standard deviation in parent age at offspring birth	8.0yr
μ_e	Mean age at interaction network expansion	18.0yr
σ_e	Standard deviation in age at interaction network expansion	4.0yr
μ_d	Mean age at death	60.0yr
σ_d	Standard deviation in age at death	12.0yr
μ_i	Mean number of parent interlocutors inherited	3.0
μ_i'	Mean number of interlocutors following expansion	10.0
h	Age homophily at network expansion	0.2
w_0	Inherited interlocutor weight at expansion	1.0
μ_R	Mean interaction rate at birth	1.0yr^{-1}
σ_R	Standard deviation in interaction rate at birth	0.1yr^{-1}
μ_r	Mean interaction rate decrease with age	10.0
σ_r	Standard deviation in interaction rate decrease with age	10.0
μ_θ	Mean time over which interaction rate decays	20.0yr

Parameter	Meaning	Value
σ_θ	Standard deviation in time over which interaction rate decays	10.0y
q	Probability each interlocutor participates in an interaction	0.5
μ_ϵ	Mean fraction of innovation frequency that is replaced	0.15
σ_ϵ	Standard deviation in fraction of innovation frequency that is replaced	0.15

Table S1.5: Parameter values in the demonstration agent-based model

Moreover, we can form naive estimates of the parameters T_M, χ and N_e by averaging over the distributions set out above. For example, we can calculate the mean interaction rate $\overline{R}$ by averaging over the distributions of speaker lifetimes and the parameters R_0, R_∞ and θ that govern how an individual's interaction rate changes over time. We estimate

$$T_M = \frac{1}{\overline{R\epsilon}} \quad , \quad s = \overline{\chi} \quad \text{and} \quad N_e = \overline{N}\frac{\overline{\epsilon}}{\overline{\epsilon^2}} \; . \tag{S1.24}$$

The resulting parabolas are plotted as solid lines of Figure S1.4. We find that the amplitude of the second jump moment (which characterises the stochastic contribution to the dynamics) is well-described by this estimate, whilst that of first jump moment is of the right order of magnitude but is over-estimated. This demonstrates that the additional complexity of this model does not fundamentally change its behaviour, but instead leads to values of the parameters in the Wright-Fisher (and therewith, the origin-fixation) model that deviate slightly from estimates obtained by simple averaging.

This observation has two important consequences for our analysis. First, by surveying all combinations of the parameters T_M, s and N_e, we ultimately account for any model which—like the demonstration model here—falls into the large Wright-Fisher class. Second, in addition to the Wright-Fisher model providing a robust description of many different evolutionary processes, the interpretation of quantities like memory lifetime and individual biases given in the main text also generalises beyond the specific individual-based model presented there.

References

[1] Traugott E. Syntax. In: Hogg RM, editor. The Cambridge History of the English Language, Vol. 1: The beginnings to 1066. Cambridge: Cambridge University Press; 1992. p. 168–289.

[2] Fischer O. Syntax. In: Blake N, editor. The Cambridge History of the English Language, Vol. 2: 1066–1476. Cambridge: Cambridge University Press; 1992. p. 207–408.

[3] Harbert W. The Germanic Languages. Cambridge: Cambridge University Press; 2007.

[4] Keller RE. The German Language. New Jersey: Humanities Press; 1978.

[5] Haugen E. Scandinavian Language Structures: A Comparative Historical Survey. Minneapolis: University of Minnesota Press; 1982.

[6] Holmes P, Hinchliffe I. Swedish: A Comprehensive Grammar. London: Routledge; 1994.

[7] Thurneysen R. A Grammar of Old Irish. Dublin: School of Celtic Studies, Dublin Institute of Advanced Studies; 1946.

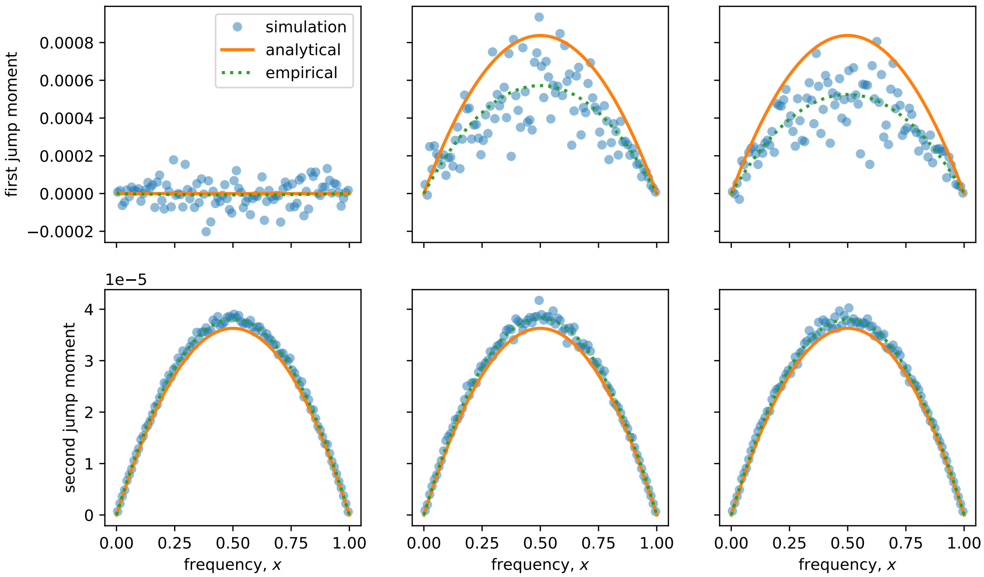

Figure S1.4: Jump moments $\overline{\Delta x}$ and $\overline{\Delta x^2}$ obtained from simulations (points) of the demonstration model over time intervals of $\Delta t = 10$yr. Dashed lines are empirical fits to the parabolic form $Ax(1-x)$ that corresponds to a Wright-Fisher model, where A is a free parameter. Solid lines correspond to amplitudes A obtained analytically through naive averaging of the relevant quantities in the demonstration model. The second jump moments are well described by these naive estimates, whilst first jump moments have the desired parabolic with an amplitude of the expected order of magnitude.

[8] Dillon M, ó Cróinín D. Irish. London: English Universities Press; 1961.

[9] Evans DS. A Grammar of Middle Welsh. The Dublin Institute for Advanced Studies; 1976.

[10] King G. Modern Welsh: A Comprehensive Grammar. 2nd ed. London: Routledge; 2003.

[11] Goodwin WW. A Greek Grammar. Boston: Ginn; 1892.

[12] Smyth HW. Greek Grammar. Cambridge, Mass.: Harvard University Press; 1920.

[13] Horrocks G. Greek: A History of the Language and its Speakers. Chichester: Wiley-Blackwell; 2010.

[14] Holton D, Mackridge P, Philippaki-Warburton I. Greek: A Comprehensive Grammar of the Modern Language. London: Routledge; 1997.

[15] Clackson J, Horrocks G. The Blackwell History of the Latin Language. Chichester: Wiley-Blackwell; 2007.

[16] Price G. The French Language: Present and Past. London: Edward Arnold; 1971.

[17] Maiden M. A Linguistic History of Italian. London: Longmans; 1995.

[18] Bourciez É. Éléments de linguistique romane. Paris: C. Klincksieck; 1956.

[19] Huntley D. Old Church Slavonic. In: Comrie B, Corbett GG, editors. The Slavonic Languages. London: Routledge; 1993. p. 125–87.

[20] Scatton EA. Bulgarian. In: Comrie B, Corbett GG, editors. The Slavonic languages. London: Routledge; 1993. p. 188–248.

[21] Timberlake A. A Reference Grammar of Russian. Cambridge: Cambridge University Press; 2004.

[22] Loprieno A. Ancient Egyptian: A Linguistic Introduction. Cambridge: Cambridge University Press; 1995.

[23] Allen JP. The Ancient Egyptian Language: An Historical Study. Cambridge: Cambridge University Press; 2010.

[24] Junge F. Late Egyptian Grammar: An Introduction. 2nd ed. Oxford: Griffith Institute; 2005.

[25] Holes C. Modern Arabic: Structures, Functions, Varieties. London: Longmans; 1995.

[26] Lambdin TQ. Introduction to Biblical Hebrew. New York: Charles Scribner's Sons; 1971.

[27] Glinert L. The Grammar of Modern Hebrew. Cambridge: Cambridge University Press; 1989.

[28] Coffin EA, Bolozky S. A Reference Grammar of Modern Hebrew. Cambridge: Cambridge University Press; 2005.

[29] Skjærvø PO. An Introduction to Old Persian; 2005.

[30] Skjærvø PO. Old Iranian. In: Windfuhr G, editor. The Iranian Languages. London: Routledge; 2009. p. 43–195.

[31] Lazard G. A Grammar of Contemporary Persian. Costa Mesa, CA: Mazda Publishers; 1992.

[32] Masica CF. The Indo-Aryan Languages. Cambridge: Cambridge University Press; 1991.

[33] Sohn HM. The Korean Language. Cambridge: Cambridge University Press; 1999.

[34] Frellesvig B. A History of the Japanese Language. Cambridge: Cambridge University Press; 2010.

[35] Norman J. Chinese. Cambridge: Cambridge University Press; 1988.

[36] Launey M. Introduction à la langue et à la littérature aztèques. Paris: L'Harmattan; 1981.

[37] Sullivan T. Compendium of Nahuatl Grammar. Miller WR, Dakin K, editors. Salt Lake City: University of Utah Press; 1988.

[38] Tuggy DH. Tetelcingo Nahuatl. In: Langacker RW, editor. Studies in Uto-Aztecan Grammar, Vol. 2: Modern Aztec Grammatical Sketches. Arlington: Summer Institute of Linguistics and The University of Texas at Arlington; 1979. p. 1–140.

[39] Brockway E. North Puebla Nahuatl. In: Langacker RW, editor. Studies in Uto-Aztecan Grammar, Vol. 2: Modern Aztec Grammatical Sketches. Arlington, Texas: Summer Institute of Linguistics and The University of Texas at Arlington; 1979. p. 141–98.

[40] Beller R, Beller P. Huasteca Nahuatl. In: Langacker RW, editor. Studies in Uto-Aztecan Grammar, Vol. 2: Modern Aztec Grammatical Sketches. Arlington, Texas: Summer Institute of Linguistics and The University of Texas at Arlington; 1979. p. 199–306.

[41] Sischo WR. Michoacán Nahuatl. In: Langacker RW, editor. Studies in Uto-Aztecan Grammar, Vol. 2: Modern Aztec Grammatical Sketches. Arlington, Texas: Summer Institute of Linguistics and The University of Texas at Arlington; 1979. p. 307–380.

[42] McQuown NA. Classical Yucatec (Maya). In: McQuown NA, editor. Linguistics. vol. 5 of Handbook of Middle American Indians. Austin: University of Texas Press; 1967. p. 201–47.

[43] Bolles D, Bolles A. A Grammar of the Yucatecan Mayan Language. Revised ed. Los Angeles: Foundation for the Advancement of Mesoamerican Studies.; 1996.

[44] Edmonson MS. Classical Quiché. In: McQuown NA, editor. Handbook of Middle American Indians, Vol. 5: Linguistics. Austin: University of Texas Press; 1967. p. 249–67.

[45] López Ixcoy CDS. Ri ukemiik ri K'ichee' chi': gramática K'ichee'. Guatemala, Guatemala: Cholsamaj; 1997.

[46] Maxwell JM, Hill RM. Kaqchikel Chronicles: The Definitive Edition. Austin: University of Texas Press; 2006.

[47] Brown RM, Maxwell JM, Little WE. ¿La ütz awäch? Introduction to Kaqchikel Maya Language. Austin: University of Texas Press; 2006.

[48] Fähnrich H. Old Georgian. In: Harris AC, editor. The Kartvelian Languages. vol. 1 of The Indigenous Languages of the Caucasus. Delmar, NY: Caravan Books; 1991. p. 129–217.

[49] Tuite K. Early Georgian. In: Woodard R, editor. Encyclopedia of the World's Ancient Languages. Cambridge: Cambridge University Press; 2004. p. 967–87.

[50] Hewitt BG. Georgian: A Structural Reference Grammar. Amsterdam: John Benjamins; 1995.

[51] Clackson JPT. Classical Armenian. In: Woodard R, editor. Encyclopedia of the World's Ancient Languages. Cambridge: Cambridge University Press; 2004. p. 922–42.

[52] Dum-Tragut J. Armenian: Modern Eastern Armenian. Amsterdam: John Benjamins; 2009.

[53] Creason S. Aramaic. In: Woodard R, editor. Encyclopedia of the World's Ancient Languages. Cambridge: Cambridge University Press; 2004. p. 381–426.

[54] Lambdin TO. Introduction to Classical Ethiopic (Ge'ez). Missoula, Mont.: Scholars Press; 1978.

[55] Gragg G. Ge'ez (Ethiopic). In: Hetzron R, editor. The Semitic Languages. London: Routledge; 1997. p. 242–60.

[56] Faber A. Genetic subgrouping of the Semitic languages. In: Hetzron R, editor. The Semitic Languages. London: Routledge; 1997. p. 3–15.

[57] Kogan LE. Tigré. In: Hetzron R, editor. The Semitic Languages. London: Routledge; 1997. p. 446–56.

[58] Raz S. Tigre Grammar and Texts. vol. 4 of Afroasiatic Dialects. Malibu, Calif: Undena Press; 1983.

[59] Huehnergard J, Woods C. Akkadian and Eblaite. In: Woodard R, editor. Encyclopedia of the World's Ancient Languages. Cambridge: Cambridge University Press; 2004. p. 218–87.

[60] Moscati S, Spitaler A, Ullendorff E, von Soden W. An Introduction to the Comparative Grammar of the Semitic Languages: Phonology and Morphology. Wiesbaden: Otto Harassowitz; 1980.

[61] Michalowski P. Sumerian. In: Woodard R, editor. Encyclopedia of the World's Ancient Languages. Cambridge: Cambridge University Press; 2004. p. 19–59.

[62] Jagersma AH. A Descriptive Grammar of Sumerian. Universiteit Leiden; 2010.

[63] Steever SB. Old Tamil. In: Woodard R, editor. Encyclopedia of the World's Ancient Languages. Cambridge: Cambridge University Press; 2004. p. 1015–40.

[64] Rajam VS. A Reference Grammar of Classical Tamil Poetry. Philadelphia: American Philosophical Society; 1992.

[65] Asher RE. Tamil. Croom Helm Descriptive Grammars. London: Croom Helm; 1982/1985.

[66] Caldwell R. A Comparative Grammar of the Dravidian or South-Indian Family of Languages. Madras: University of Madras; 1875/1961.

[67] Beyer SV. The Classical Tibetan Language. State University of New York Press; 1992.

[68] DeLancey S. Classical Tibetan. In: Thurgood G, LaPolla RJ, editors. The Sino-Tibetan Languages. London: Routledge; 2003. p. 253–69.

[69] Denwood P. Tibetan. Amsterdam: John Benjamins; 1999.

[70] Poppe N. Grammar of Written Mongolian. Wiesbaden: Otto Harrassowitz; 1974.

[71] Binnick RI. Modern Mongolian: A Transformational Syntax. Toronto: University of Toronto Press; 1979.

[72] Kerslake C. Ottoman Turkish. In: Johanson L, Csató ÉÁ, editors. The Turkic Languages. London: Routledge; 1998. p. 179–202.

[73] Lewis GL. Turkish Grammar. Oxford: Oxford University Press; 1967.

[74] Sidwell P. Classifying the Austroasiatic Languages: History and State of the Art. Munich: Lincom Europa; 2009.

[75] Jenner PN, Sidwell P. Old Khmer Grammar. vol. 611 of Pacific Linguistics. Canberra: The Australian National University; 2010.

[76] Maspero G. Grammaire de la langue khmère (cambodgien). Paris: Imprimerie Nationale; 1915.

[77] Adelaar WFH, Muysken PC. The Languages of the Andes. Cambridge: Cambridge University Press; 2004.

[78] Parker G. Ayacucho Quechua Grammar and Dictionary. The Hague: Mouton; 1968.

[79] Cole P. Imbabura Quechua. London: Croom Helm; 1985.

[80] Weber DJ. A Grammar of Huallaga (Huánaco) Quechua. vol. 112 of University of California Publications in Linguistics. Berkeley: University of California Press; 1989.

[81] Bertonio PL. Arte y grammatica muy copiosa de la lengua aymara. Rome: Luis Zanetti; 1603.

[82] Hardman MJ. Aymara. Munich: Lincom Europa; 2001.

[83] de Valdivia L. Arte y gramatica general de la lengua que corre in todo el Reyno de Chile, con un vocabulario, y confessionario. Lima: Francisco del Canto; 1060.

[84] Smeets I. A Grammar of Mapuche. vol. 41 of Mouton Grammar Library. Berlin: Mouton de Gruyter; 2008.

[85] McEvedy C, Jones R. Atlas of World Population History. Harmondsworth: Penguin; 1978.

[86] Grimes BF. Ethnologue. 11th ed. Summer Institute of Linguistics; 1988.

[87] McCandlish DM, Stoltzfus A. Modeling evolution using the probability of fixation: History and implications. The Quarterly Review of Biology. 2014;89:225–52.

[88] Crow JF, Kimura M. An Introduction to Population Genetics Theory. New York: Harper and Row; 1970.

[89] Abate J, Whitt W. A Unified Framework for Numerically Inverting Laplace Transforms. INFORMS Journal on Computing. 2006;18:408–21.

[90] Blythe RA. Source code and sample datasets; 2021. `https://git.ecdf.ed.ac.uk/rblythe3/gram-cycles`.

[91] Sood V, Redner S. Voter Model on Heterogeneous Graphs. Phy Rev Lett. 2005;94:178701.

[92] Antal T, Redner S, Sood V. Evolutionary dynamics on degree-heterogeneous graphs. Physical Review Letters. 2006;96:188104.

[93] Baxter GJ, Blythe RA, McKane AJ. Fixation and consensus times on a network: A unified approach. Physical Review Letters. 2008;101:258701.

[94] Kimura M, Ohta T. The average number of generations until fixation of a mutant gene in a finite population. Genetics. 1969;61:763–71.

[95] Nordborg M. Coalescent theory. In: Handbook of Statistical Genomics. John Wiley; 2019. p. 145.
